# Hidden diversity at the edges of maps: morphometrics of *Carex* sect. *Uncinia* (Cyperaceae) helps unravel taxonomic diversity in subantarctic and remote archipelagos

**DOI:** 10.3897/phytokeys.277.189029

**Published:** 2026-07-21

**Authors:** Pablo García-Moro, Raúl Lois, Kerry A. Ford, Isabel Larridon, Germinal Rouhan, Eric H. Roalson, Pedro Jiménez-Mejías

**Affiliations:** 1 Área de Botánica, Departamento de Biología Molecular e Ingeniería Bioquímica, Universidad Pablo de Olavide, Av. Rectora Rosario Valpuesta Fernández, 1, 41089, Dos Hermanas, Sevilla, Spain Royal Botanic Gardens, Kew Richmond United Kingdom; 2 Departamento de Biodiversidad y Gestión Ambiental (Botánica), Facultad de Ciencias Biológicas y Ambientales, Universidad de León, Campus de Vegazana s/n, 24007, León, Spain Institut de Systématique, Evolution, Biodiversité (ISYEB), Muséum national d’Histoire naturelle, CNRS, Sorbonne Université, EPHE, Université des Antilles Paris France; 3 Allan Herbarium, BSI, 76 Gerald Street, Lincoln 7608, New Zealand Facultad de Ciencias Biológicas y Ambientales, Universidad de León León Spain; 4 Royal Botanic Gardens, Kew, Richmond, TW9 3AE, UK Departamento de Biología Molecular e Ingeniería Bioquímica, Universidad Pablo de Olavide Sevilla Spain; 5 Institut de Systématique, Evolution, Biodiversité (ISYEB), Muséum national d’Histoire naturelle, CNRS, Sorbonne Université, EPHE, Université des Antilles, 57 Rue Cuvier, 75005 Paris, France School of Biological Sciences, Washington State University Pullman United States of America; 6 School of Biological Sciences, Washington State University, P.O. Box 644236, Pullman, Washington 99164-4236, USA Allan Herbarium, BSI Lincoln New Zealand

**Keywords:** Herbarium vouchers, insular speciation, Pacific region, species delimitation, subantarctic islands, taxonomy, *
Uncinia
*

## Abstract

Subantarctic islands constitute some of the most remote terrestrial environments on Earth and represent the southernmost limits of non-glaciated land. Despite their comparatively low vascular plant diversity, several taxa are reported as widely distributed across multiple archipelagos without detailed comparative taxonomic assessment. In *Carex* sect. *Uncinia* (Cyperaceae), the broadly circumscribed *C. brevicaulis* and *C. austrocompacta* complexes, have long been assumed to represent single species with wide and disjunct distributions across remote oceanic islands far from continental landmasses. In this study, a comparative morphometric approach was employed to reassess species limits within these complexes across subantarctic and Pacific archipelagos. Multivariate analyses revealed consistent morphological discontinuities corresponding to geographically structured lineages. The results support the recognition of four distinct species within the *C. brevicaulis* complex and five within the subantarctic *C. austrocompacta* complex, some of them newly described here. These findings reveal substantial hidden diversity in oceanic *Carex* and demonstrate the effectiveness of quantitative morphology in resolving taxonomic complexity in morphologically reduced groups.

## Introduction

Subantarctic islands are at the southernmost limits of non-glaciated land and, therefore, the edge of the vascular flora realm. These islands were placed by Takhtajan (1986) in the Antarctic kingdom, together with southern Patagonia and the southern islands of New Zealand. A total of 886 vascular plant species have been recorded as present in these archipelagos, 58.62% of them considered native and only 9.61% endemic (Guerrero et al. 2025). Despite these relatively poor levels of diversity, comparative studies on the taxonomy of plants from subantarctic islands are virtually nonexistent. A number of subantarctic species are reported to be shared among different archipelagos (Guerrero et al. 2025; POWO 2026), despite the lack of comparative studies between the populations of these species across the different island systems. A recent biogeographic study on several subantarctic species (Aguado-Lara et al. 2025) revealed that several of the species distributed among different archipelagos form monophyletic groups, with divergence ages of the same species between island groups dating back to the end of the Pliocene or the first half of the Pleistocene. Such levels of divergence typically involve lineages recognized as different species in other plant groups.

**Figure 1.**
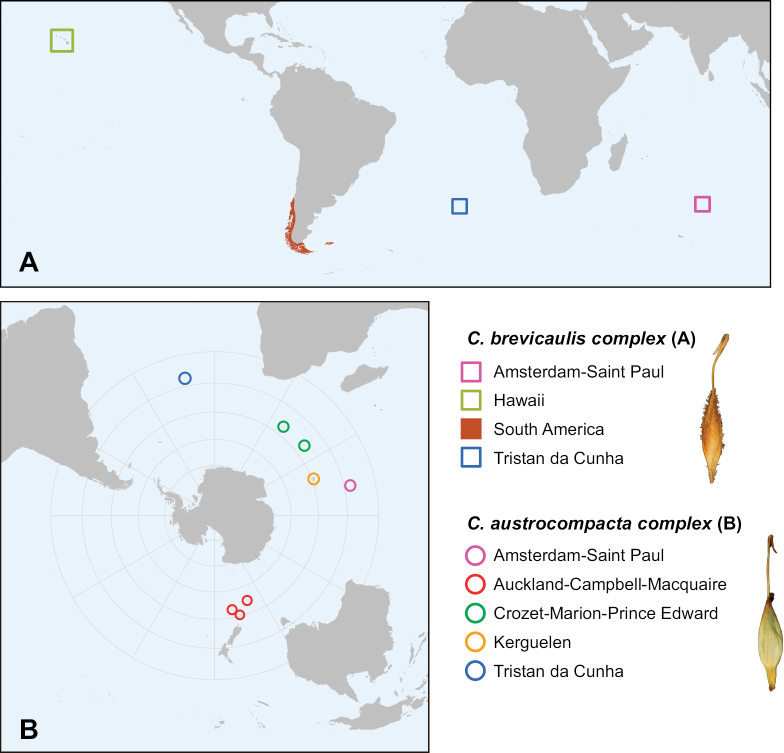
Distribution maps of the specimens analyzed for *Carex brevicaulis* complex (**A**), subantarctic *Carex austrocompacta* complex (**B**).

The genus *Carex* L. (Cyperaceae) comprises over 2,000 accepted species, ranking among the five largest genera of angiosperms (Roalson et al. 2021; POWO 2026). It has a cosmopolitan distribution but is primarily diversified in the Northern Hemisphere (Martín-Bravo et al. 2019). Among the six subgenera of the genus, the subgenus *Uncinia* has been shown to constitute a unique radiation in the Southern Hemisphere. Its largest section, sect. *Uncinia* displays a highly disjunct distribution between South America and the southwestern Pacific while also being present in a number of oceanic archipelagos, including several subantarctic island groups (García-Moro et al. 2022). Among the species distributed on islands, two taxa have been reported as strikingly widely distributed: *C. brevicaulis* Thouars and *C. austrocompacta* K.L.Wilson (POWO 2026; Fig. 1).

*Carex brevicaulis* Thouars was first described in 1808 in an illustrated book for the flora of Tristan da Cunha (du Petit-Thouars 1808, 1811). The name was simultaneously published with an alternative name under *Uncinia* (*U. brevicaulis* Thouars) and treated as such until the reincorporation of *Uncinia* within *Carex* (Global Carex Group 2015). Under the name *C. brevicaulis*, populations have been reported from different regions, including localities in South America (Kükenthal 1909; Moore 1968; Barros 1969), circum-Antarctic archipelagos (Amsterdam-Saint Paul and Tristan da Cunha; Kükenthal 1909), and the Pacific archipelago of Hawaii (Henrickson and Herbst 1988) (Fig. 1). Among these sets of populations, only the identity of the South American plants has raised some discussion, with authors differently treating it as a variety (*U. brevicaulis* var. macloviana (Gaudich.) Kük.; Kükenthal 1909; Barros 1969) or even at species rank as *U. macloviana* Gaudich (= *C. delacosta* Kuntze) (Wheeler 2007). The identity of the other island populations identified as *C. brevicaulis* has never been debated.

**Figure 2.**
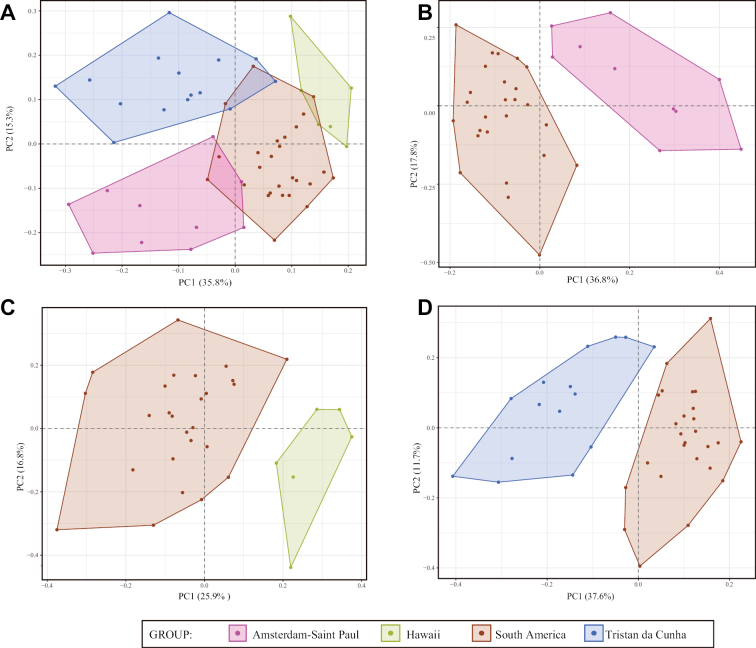
Principal component analyses (PCA) of the *Carex brevicaulis* complex. **A**. PCA plot of the whole complex; **B**. PCA comparing South America vs. Amsterdam–Saint Paul; **C**. PCA comparing South America vs. Hawaii; **D**. PCA comparing South America vs. Tristan da Cunha. Axis values represent the percentage of variance explained by PC1 and PC2.

*Carex austrocompacta* K.L.Wilson was a name created to accommodate *Uncinia compacta* R.Br. in *Carex*, a taxon described from Tasmania (Brown 1810). The name was later used to refer to populations of *Carex* sect. *Uncinia* taxa from different subantarctic archipelagos: Tristan da Cunha in the southern Atlantic, Marion-Prince Edward, Crozet, Amsterdam-Saint Paul, and Kerguelen in the southern Indian Ocean, and Macquarie, Auckland, and Campbell islands in the southwestern Pacific (POWO 2026; Fig. 1). In addition, up to four additional related names have been reported from these island systems: (1) *Uncinia hookeri* Boott (*C. erebus* K.A.Ford) (Hooker 1844), described from Auckland and Campbell islands and later reported from Macquarie and Antipodes (Moore and Edgar 1970) and Stewart Island (Lehnebach 2010); (2) *Uncinia moseleyana* Boeckeler (Boeckeler 1878), from Kerguelen; (3) *Uncinia dikei* Nelmes (*C. dikei* (Nelmes) K.L.Wilson), from Marion Island (Nelmes 1949; Gremmen 1981); and (4) *U. hookeri* var. *elongata* (C.B.Clarke) Hamlin, from Amsterdam Island (Hamlin 1963). No proper comparison of these four taxa with the subantarctic populations assigned to *C. austrocompacta* exists. These names have variously persisted since in the literature, sometimes coexisting on the same island with the widely distributed reports of *C. austrocompacta* (POWO 2026) (Fig. 2).

In this paper, the aim is to revise the identity of the island populations ascribed to *C. brevicaulis* and *C. austrocompacta*, with special emphasis on the subantarctic archipelagos, to elucidate whether the populations in some of the islands may be recognized as independent species and to properly describe them when pertinent.

## Materials and methods

### Delimitation of the study group and sampling

The study focused separately on the two species complexes: the *Carex brevicaulis* complex and the subantarctic populations assigned to the *C. austrocompacta* complex (hereafter the subantarctic *C. austrocompacta* complex). Material was obtained on loan from the BISH, BM, K, NY, P, and US herbaria (herbarium codes according to Thiers (2026); Suppl. material 3: table SS1). The sampling from the different archipelagos may be limited due to the low number of collections from these regions. However, a concerted effort was made to locate and examine the maximum number of specimens for each regio.

For the *Carex brevicaulis* complex, the populations from Tristan da Cunha, Amsterdam-Saint Paul, and Hawaii were considered separate groups. Populations from mainland South America and the Falkland Islands were also included as representatives of the continental taxon, currently recognized as *C. delacosta*, whose taxonomic distinctness from insular populations has long been debated. A total of 57 specimens from across the regions were studied: six specimens from the Hawaii archipelago, 10 specimens from Amsterdam-Saint Paul, 15 from Tristan da Cunha, and 28 from South America and the Falklands.

For the subantarctic *Carex austrocompacta* complex (including *C. erebus*, *U. moseleyana*, *C. dikei*, and *U. hookeri* var. *elongata*), the populations from each archipelago were again considered separate groups (west to east from the Atlantic to the Pacific): Tristan da Cunha, Marion-Prince Edward, Crozet, Kerguelen, Amsterdam-Saint Paul, and the subantarctic archipelagos of the southwestern Pacific. Paradoxically, in this case, *C. austrocompacta* itself was not considered, as preliminary work, detected using the available keys, determined that this is a temperate taxon restricted to Australia and Tasmania and that its citation in subantarctic archipelagos was a misuse of the name. A total of 55 specimens were studied: 24 from Tristan da Cunha, four from Marion-Prince Edward, 12 from Crozet, five from Kerguelen, seven specimens from Amsterdam-Saint Paul, and three from the subantarctic southwestern Pacific islands of Macquarie and Campbell.

### Statistical analyses for a morphometric approach

Morphometric analyses were conducted to explore patterns of morphological variation and to test whether predefined groups could be statistically distinguished based on quantitative characters. As explained above, the populations of each set of islands were initially considered different groups.

A different set of quantitative morphological characters was selected for each complex (Table 1) to capture variation across vegetative and reproductive structures: 20 characters for the *C. brevicaulis* complex and 25 for the subantarctic *C. austrocompacta* complex. Characters were selected according to those reported as of taxonomic importance in *Carex* in general and in sect. *Uncinia* in particular (Kükenthal 1909; Moore and Edgar 1970; Wheeler 2007; Jiménez-Mejías and Dorr 2018; Ridley and Jiménez-Mejías 2022), plus a few more selected from the present observations.

**Table 1.** List of all variables included in the morphometric analyses for each of the species complexes considered. Glume measurements refer to pistillate glumes. All reproductive structures refer to the middle part of the spike.

Character abbreviation	Description	*C. brevicaulis*	*C. austrocompacta*
st_l	Stem length (cm)	✓	✓
st_w	Stem width at its middle part (mm)	✓	✓
lf_l	Leaf length (cm)	✓	✓
lfu_w	Uppermost leaf width (mm)	✓	✓
lfw_w	Widest leaf width (mm)	✓	✓
sp_l	Spike length (cm)	✓	✓
spm_l	Staminate potion of the spike length (mm)	✓	✓
sp_w	Spike width at its widest point (mm)	✓	✓
gl_l	Glume length (mm)	✓	✓
gl_w	Glume width at its widest point (mm)	✓	✓
gl_lbw	Glume length from base to the widest point (mm)	✓	✓
gl_wwt	Glume width at half-length from the widest point to the tip (mm)	✓	✓
ut_l	Utricle length (mm)	✓	✓
ut_w	Utricle width at its widest point (mm)	✓	✓
ut_lbw	Utricle length from base to the widest point (mm)	✓	✓
ut_wwt	Utricle width at its widest point (mm)	–	✓
ut_dep	Utricle empty distal portion (mm)	✓	✓
ut_cpl	Utricle stipitate base length (mm)	–	✓
ut_cpw	Utricle stipitate base width (mm)	–	✓
ac_l	Achene length (mm)	✓	✓
ac_w	Achene width at its widest point (mm)	✓	✓
ac_lbw	Achene length from base to the widest point (mm)	✓	✓
ac_wwt	Achene width at half-length from the widest point to the tip (mm)	–	✓
ra_l	Exserted portion of the rachilla length (mm)	✓	✓
ho_l	Rachilla hook length (mm)	–	✓

Principal component analysis (PCA) was used as an exploratory tool to investigate the structure of morphological variation within each complex. A nested-PCA approach was followed to evaluate whether distinct morphogroups could be identified within the complex, as successfully used in *Carex* (Míguez et al. 2018; Lois et al. 2025). Accordingly, in each analysis, independent morphogroups were intended to be identified. Whenever one of the resulting morphogroups contained samples belonging to more than one of the considered populations, this group of samples was split from the main dataset and reanalyzed separately. All variables were standardized prior to analysis. No transformation of the original variables was applied, as PCA, as an exploratory tool, does not require strict assumptions of normality. In addition, avoiding transformations allowed the preservation of the original biological meaning of the variables and facilitated the interpretation of the ordination axes, following common practice in other morphometric studies of vascular plants (Valcárcel and Vargas 2010), including *Carex* (Jiménez-Mejías et al. 2012). An initial exploratory PCA was performed to assess the overall organization within each dataset and identify major axes of morphological variation. Subsequently, nested PCAs were conducted whenever morphogroups of mixed populations were detected to refine the underlying morphostructure and identify combinations of characters that best captured morphological differentiation where such existed.

Once a clear morphostructure was identified from PCAs, linear discriminant analyses (LDAs) were performed to evaluate group separation under a supervised classification framework. For LDAs, datasets were randomly partitioned into training (70% of the samples) and testing (30%) subsets, allowing model performance to be evaluated using independent data.

Univariate statistics were graphically explored using boxplots to assess ranges, dispersion, and potential overlap among groups and detect the potentially best discriminant characters for plant identification (Suppl. materials 1, 2).

All statistical analyses were performed in RStudio (R Core Team 2025) using the *dplyr* (Wickham et al. 2025), *stats* (R Core Team 2025), and *MASS* (Venables and Ripley 2002) packages. To plot the results from both PCAs and LDAs, *ggfortify* (Tang et al. 2016; Horikoshi and Tang 2018) and *ggplot2* (Wickham 2016) were used.

## Results

### Principal component analyses

Principal component analysis results are presented through the graphic representation (PCA plot) of the first two components extracted by the analysis (PC1 and PC2). In all cases, these PCs had significant eigenvalues (> 1). Values of the contributions for the PCs in each of the PCAs are provided in Suppl. material 3: table S2.

For the *C. brevicaulis* complex, a first exploratory PCA (PCA1; Fig. 2A) with all samples was performed. The first two PCs accounted for 35.8% and 15.3% of the variation, respectively. The PCA plot (Fig. 2A) revealed a clear geographic structuring of the morphological variation, with specimens from each insular system forming well-defined clusters, each corresponding to a distinct morphogroup. These insular morphogroups were arranged peripherally in the ordination space. In contrast, samples of the continental taxon, *C. delacosta*, clustered near the center of the PCA plot and showed partial overlap with each of the insular morphogroups. This observed overlap reflects the projection of multivariate variation onto a reduced ordination space when all groups are analyzed simultaneously. To further assess group differentiation, a series of nested PCAs was performed (South America vs. Amsterdam-Saint Paul, PCA2; South America vs. Hawaii, PCA3; South America vs. Tristan da Cunha, PCA4; Fig. 2B–D, respectively), contrasting continental samples against each insular morphogroup separately. In these pairwise analyses (Fig. 2B–D), the continental and each of the insular groups formed clearly separated clusters, with no overlap observed along the principal components. In all cases, samples belonging to each group were consistently distributed on opposite sides of the ordination space, confirming the morphological distinctiveness of each insular morphogroup relative to the continental taxon.

**Figure 3.**
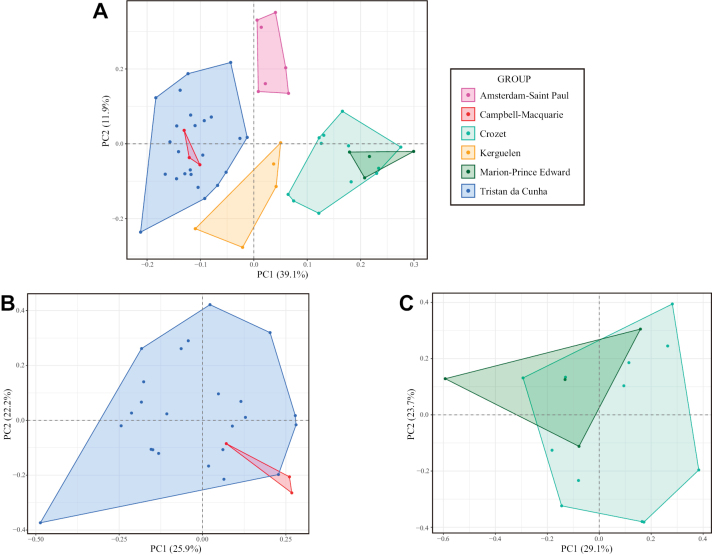
Principal component analyses (PCA) of the subantarctic *Carex austrocompacta* complex. **A**. PCA plot of the whole complex; **B**. PCA comparing Campbell–Macquarie vs. Tristan da Cunha; **C**. PCA comparing Crozet vs. Marion–Prince Edward. Axis values represent the percentage of variance explained by PC1 and PC2.

For the subantarctic *C. austrocompacta* complex, the first two PCs of the initial exploratory PCA accounted for 39.1% and 11.9% of the variation, respectively. The PCA plot (Fig. 3A) revealed four main clusters within the ordination space. Although six subantarctic island systems were included in the analysis, samples from two of these systems did not form independent clusters. Specifically, specimens from the southwestern Pacific Campbell-Macquarie populations clustered with the Tristan da Cunha samples, whereas specimens from Marion-Prince Edward clustered together with those from Crozet. In contrast, samples from Amsterdam-Saint Paul and Kerguelen each formed distinct clusters with no apparent overlap with other groups in the preliminary ordination. To further evaluate whether subtle dimensions of variation could discriminate the overlapping groups, pairwise nested PCAs contrasting Tristan da Cunha vs. Campbell-Macquarie and Crozet vs. MPE (Fig. 3B, C). In the comparison between Tristan da Cunha and Campbell-Macquarie, samples were largely separated, with only a single specimen from Macquarie showing partial overlap within the Tristan da Cunha cluster. By contrast, in the nested PCA contrasting Crozet and Marion-Prince Edward, samples from both island systems remained intermingled across the ordination space, and no clear separation between the two groups could be detected. Accordingly, in the subsequent analyses, Crozet and Marion-Prince Edward were considered a single morphogroup.

### Linear discriminant analyses

**Figure 4.**
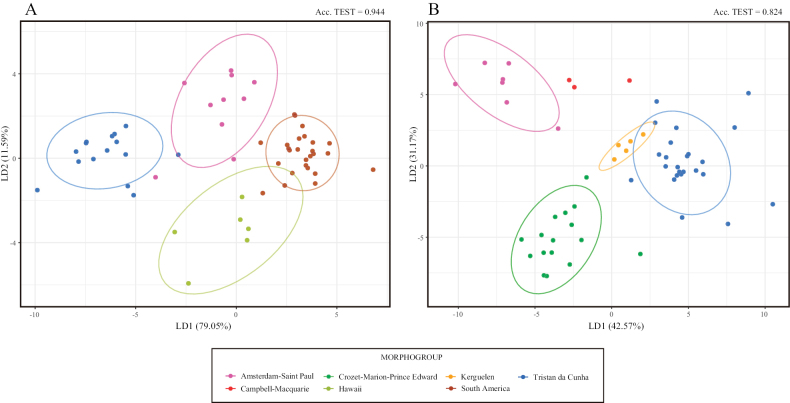
Linear discriminant analyses (LDA) of the studied complexes. **A**. *Carex brevicaulis* complex; **B**. Subantarctic *Carex austrocompacta* complex. Axis values represent the discriminant power (%) of LD1 and LD2.

Linear discriminant analysis (LDA) applied to the *C. brevicaulis* complex yielded a 94.4% correct classification of specimens, both in the training dataset and in the independent test dataset (Suppl. material 3: table S3). Almost all samples were assigned to their *a priori* defined morphogroups, with only one misclassification detected (Suppl. material 3: table S2). This result indicates very high discriminative power of the selected morphological variables and demonstrates the robustness and reliability of the LDA model (LDA1; Fig. 4A). The concordant performance obtained in both training and test datasets further suggests that the observed separation among morphogroups is not the result of overfitting but instead reflects consistent and biologically meaningful morphological differentiation within the complex. Visualization of the discriminant space defined by the first two linear discriminant functions (LD1 and LD2) revealed a clear separation among most morphogroups (Fig. 4A). Although a limited degree of overlap was observed between the Hawaii and South America morphogroups in the LD1–LD2 projection, specimens nonetheless formed well-defined and largely distinct clusters. Importantly, this partial overlap did not translate into classification errors, as all individuals were correctly assigned to their respective morphogroups in both training and test datasets. This indicates that discrimination among groups is fully resolved in the complete multivariate discriminant space, even if some proximity is observed when reduced to two dimensions.

Linear discriminant analysis applied to the *C. austrocompacta* complex resulted in a classification accuracy of 82.4% in the test dataset (Suppl. material 3: table S3). Most specimens were correctly assigned to their *a priori* defined morphogroups, although a limited number of misclassifications were detected. Specifically, one specimen of Campbell-Macquarie was not correctly classified and assigned. However, it was assigned to the Amsterdam-Saint Paul morphogroup rather than to Tristan da Cunha. Inspection of the LD1–LD2 discriminant space provides insight into the observed classification patterns (LDA2; Fig. 4B). Samples of Campbell-Macquarie form a distinct cluster clearly separated from Tristan da Cunha along the first discriminant axis, indicating that the LDA2 model is able to discriminate Campbell-Macquarie from the morphogroup in which it appeared nested in the previous PCA analysis (Fig. 4B).

Values of the contributions of the variables to the LDA models are provided in Suppl. material 3: table S4.

### Univariate statistics

The univariate boxplots are shown in Suppl. materials 1, 2.

For the *C. brevicaulis* complex, the characters that showed the least overlap were utricle width (*ut_w*) for Amsterdam-Saint Paul, utricle length (*ut_l*) and its distal empty portion (*ut_dep*) for Hawaii, glume width from its widest point to its tip (*gl_wwt*) for South America, and spike length (*sp_l*), leaf length (*lf_l*), length of the male portion of the spike (*spm_l*), and achene length (*ac_l*) for Tristan da Cunha (abbreviations according to Table 1).

For the subantarctic *C. austrocompacta* complex, the characters that showed the least overlap were length of the rachilla (*ra_l*) for Amsterdam-Saint Paul, *st_w* for Campbell-Macquarie, widest leaf width (*lfw_w*), spike width (*sp_w*), and utricle width (*ut_w*) for Crozet-Marion-Prince Edward, leaf length (*lf_l*) for Kerguelen, and utricle length (*ut_l*) and glume width from its widest point to its tip (*gl_wwt*) for Tristan da Cunha.

## Discussion

The morphometric approach revealed that, within the two studied groups, there is neglected taxonomic structure. As in previous works on other *Carex* groups (Míguez et al. 2018; Lois et al. 2025), the accurate evaluation of the taxonomic limits of the considered populations has led to the recognition of six previously unrecognized species.

The historical lack of comparison between populations of different archipelagos, despite the long-known occurrence of these taxa in some of them, can be attributed to several factors. Only Hamlin (1963) performed a detailed comparison between plants from different archipelagos, but based on very few collections. First, the different archipelagos are under different political administrations. In this sense, Hawaii is an American state, Tristan da Cunha is a UK Overseas Territory, Marion-Prince Edward belongs to South Africa, the Crozet, Amsterdam, Saint Paul, and Kerguelen archipelagos are French Southern Territories, Macquarie Island is administered by Australia, and the Campbell, Auckland, and Antipodes islands by New Zealand. Such different administrative situations lead to the deposition of collections in different herbaria, sometimes in remote parts of the world (see Thiers 2026), which makes direct comparison between populations from the cited archipelagos difficult. Second, each of these islands is remote and, in most cases, difficult to access. Apart from Hawaii, only Tristan da Cunha harbors a permanent non-scientific population. This makes the available collections scarce and fragmentary, which hampers taxonomic studies. Lastly, the taxonomy of *Carex* in general and that of sect. *Uncinia* in particular is intricate. The extreme reduction of the taxonomically relevant parts masks possible discriminant characters to the untrained eye. Indeed, it was not until the recent advance in the knowledge of the genus in the Southern Hemisphere that several previously unnoticed species were identified and formally described (Jiménez-Mejías et al. 2021; Ford 2025; Morales-Alonso et al. 2025).

According to the results, each of the detected morphogroups is recognized as an independent taxon at species rank given its morphological distinctiveness. Four different species are recognized within the *C. brevicaulis* complex (Amsterdam-Saint Paul, Hawaii, Tristan da Cunha, plus the South American populations recognized as *C. delacosta*) and five for the subantarctic *C. austrocompacta* complex (Amsterdam-Saint Paul, Campbell-Macquarie, Crozet-Marion-Prince Edward, Kerguelen, and Tristan da Cunha). These results are in agreement with phylogenomic reconstructions (García-Moro et al. unpubl. data) that show that each of these groups constitutes an independent lineage. This indeed reinforces the consideration of the subantarctic southwestern Pacific plants, represented here by the few Campbell-Macquarie samples, as a distinct taxon despite their seeming overlap in the PCA explorations (Fig. 3A, B).

The following taxonomic treatment and identification key are presented below.

### Taxonomic treatment

#### *Carex brevicaulis* group

**Table:** 

1	Utricles 4.0–4.4 mm long, with a distal empty portion 0.7–1.0 mm long; spikes 3.0–5.6 cm long; plants from Hawaii	***C. haleakalensis***
–	Utricles 4.3–6.6 mm long, with a distal empty portion 1.1–2.2 mm long; spikes 4.0–16.5(18.0) cm long	**2**
2	Pistillate scales 3.8–5.2 mm; spikes 2.1–4.5 mm at widest; plants from southern South America and the Falklands	***C. delacosta***
–	Pistillate scales 5.2–9.8 mm; spike 3.8–8.6 mm at widest	**3**
3	Spikes 3.0–9.5 cm long, with a 4.0–13.0 cm long staminate part; longest leaves 6–35 cm long; plants from Amsterdam and Saint Paul islands	***C. donjuanii***
–	Spikes 8.0–18.0 cm long, with a 7.4–25.0 cm long staminate part; longest leaves 30–76 cm long; plants from Tristan da Cunha archipelago	***C. brevicaulis***

##### Carex brevicaulis

Taxon classificationPlantaePoalesCyperaceae

Thouars, Esquisse Fl. Tristan d’Acugna: 35 (1808).

427BCE49-C955-5528-9F2B-453D76857D65

Figs 5A, 6A

≡ Uncinia brevicaulis Thouars, Esquisse Fl. Tristan d’Acugna: 35 (1808). Lectotype (**here designated**). Tristan d’Acugna • *Herbier du Petit-Thouars* (P04023173!).
= Uncinia brevicaulis var. *gracilior* Hemsl., Rep. Voy. Challenger, Bot. 1(3): 160, tab. XLVI (1885). Lectotype (**here designated**). “Uncinia gracilis,” Tristan d’Acugna • *Herbier du Petit-Thouars* (P04023166!).

###### Etymology.

From the Latin *brevis*-, short, and the Greek *kaulos*, stem.

**Figure 5.**
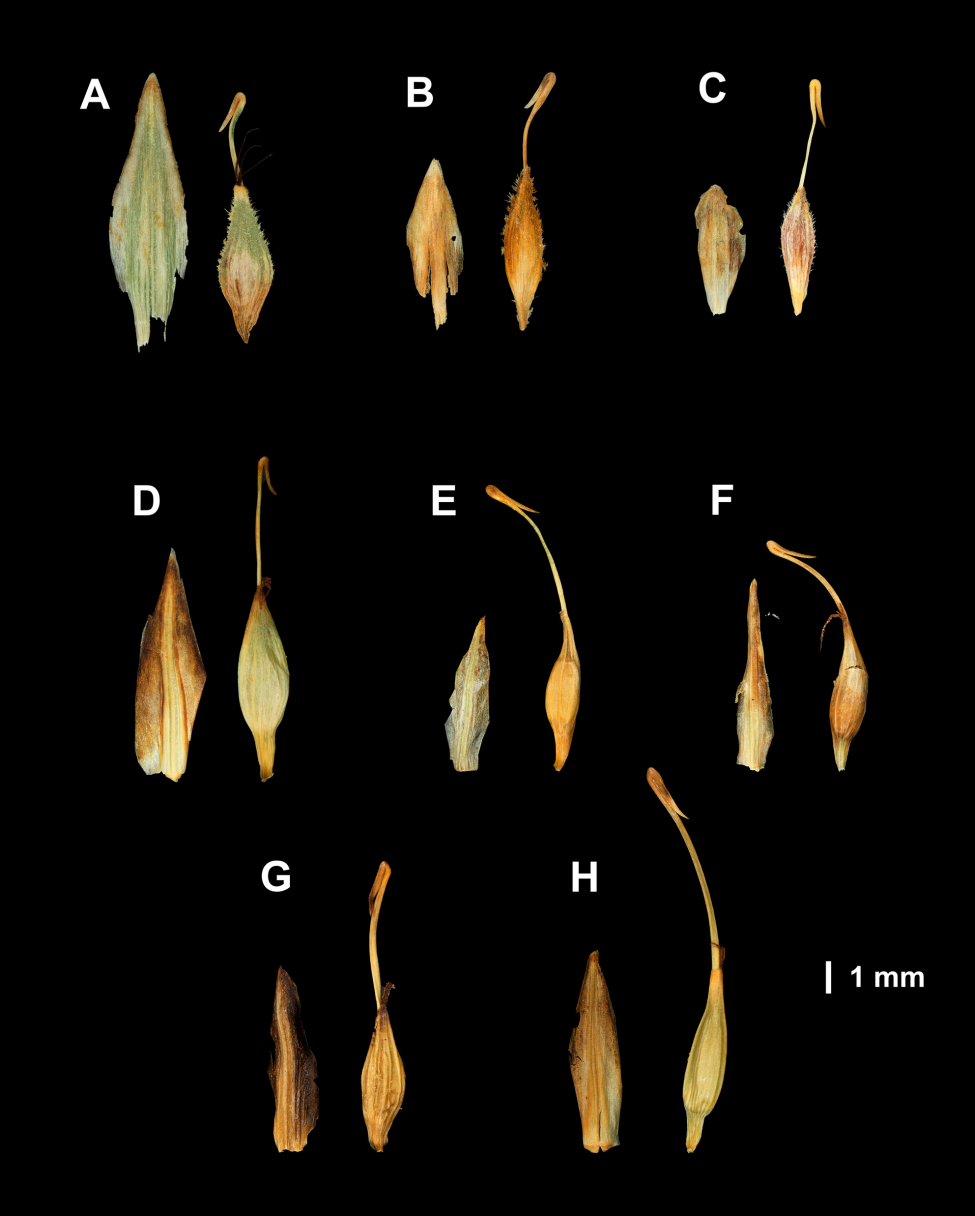
Utricles and glumes of the studied species. **A**. *Carex brevicaulis* (Tristan da Cunha, Tristan Island, *N. J. M. Gremmen T07 0495*; K000649069); **B**. *C. donjuanii* (Amsterdam Island, *P. Noël s.n*., P00790191); **C**. *C. haleakalensis* (Hawaii, Maui, *J. Henrickson and R. Vogl 3894*, US3090900, barcode 00436681); **D**. *C. dikei* (Crozet Islands, *M. Segonzac 40*, P04343722); **E**. *C. erebus* (Macquarie Island, *R. D. Seppelt 12661*, BM 013718995); **F**. *C. moro-ortegae* (Tristan da Cunha, Tristan Island, *Y. Mejland 1654*, K000298924); **G**. *C. moseleyana* (Kerguelen archipelago, *J.-C. Jolinon 749*, P04177500); **H**. *C. nao-victoriae* (Amsterdam Island, *A. Lourteig and P. Cour 41*, P04343692).

**Figure 6.**
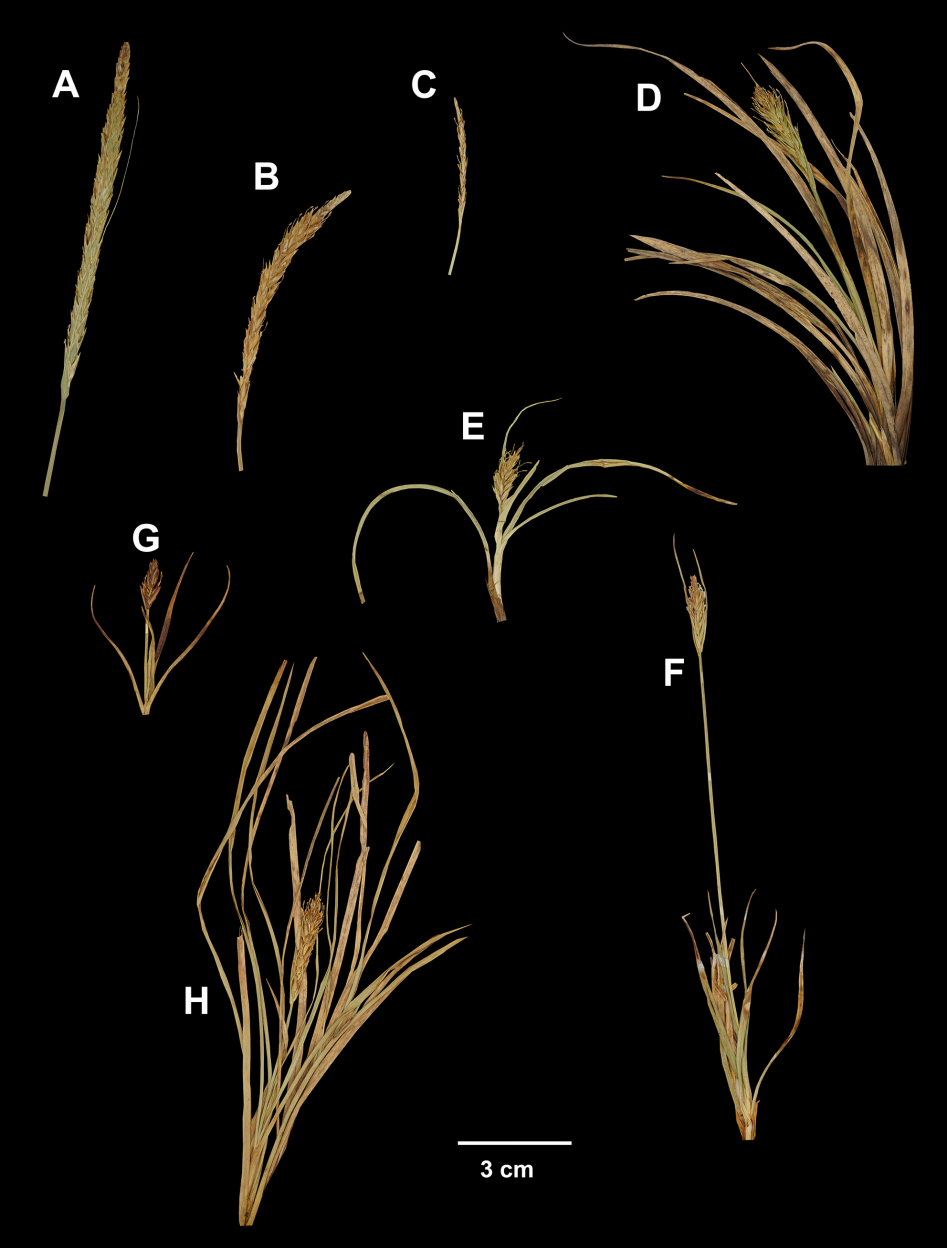
Inflorescences of the studied species. **A**. *Carex brevicaulis* (Tristan da Cunha, Tristan Island, *N. J. M. Gremmen T07 0495*; K000649069); **B**. *C. donjuanii* (Amsterdam Island, *P. Noël s.n*., P00790191); **C**. *C. haleakalensis* (Hawaii, Maui, *J. Henrickson and R. Vogl 3894*, US3090900, barcode 00436681); **D**. *C. dikei* (Crozet Islands, *T. Lefebvre 31*, P02433243); **E**. *C. erebus* (Macquarie Island, *R. D. Seppelt 12661*, BM 013718995); **F**. *C. moro-ortegae* (Tristan da Cunha, Tristan Island, *Y. Mejland 1654*, K000298924); **G**. *C. moseleyana* (Kerguelen archipelago, *J.-C. Jolinon 749*, P04177500); **H**. *C. nao-victoriae* (Amsterdam Island, *A. Lourteig and P. Cour 41*, P04343692).

###### Description.

***Plants*** laxly caespitose, with short rhizomes. ***Fertile culms*** 13.5–61.0 cm long, 0.8–2.0 mm wide, erect or slightly curved, shorter to longer than leaves, subrounded to obtusely trigonous, smooth. ***Leaves*** 30.3–64.8(76.0) cm long, the widest ones 3.5–5.7 mm wide, the uppermost leaf 1.1–5.3 mm wide, coriaceous, flat to slightly M-shaped, the margins antrorsely scabrid. ***Inflorescence*** a solitary, androgynous spike, 8–16.5(18.0) cm × 3.8–7.6 mm, bractless or occasionally the lowermost glume developing into a setaceous bract up to as long as the spike, never surpassing it, long-oblong, cylindrical, densely flowered, with usually more than 60 pistillate flowers, ripe utricles adpressed to ascendant; staminate portion 7.4–21.7(25.0) mm long. ***Pistillate glumes*** from the middle part of the spike 5.2–9.8 × 1.6–2.6 mm, with 2.0–5.2 mm from base to the widest point, 1.1–1.8 mm wide at half-length from the widest point to the tip, longer than the utricles, elliptic-oblong to fusiform, the apex long-acute, membranaceous, surface glabrous, greenish to straw-colored, occasionally brownish, paler towards the sides and also with the margins narrowly brownish, with the tip narrowly hyaline and the margins sometimes slightly scabrid, 1–2 prominent central veins, greenish, the sides several faintly veined. ***Utricles*** 4.3–5.6 × 1.3–1.8 mm, with 2.0–2.9 mm long from base to its widest point, elliptic, plano-convex, greenish to brownish, pilose with adpressed hairs on the sides more densely arranged towards the tip, the margins with spreading hairs mostly arranged into fascicles, with several conspicuous raised veins adaxially and abaxially, proximally tapered to a substipitate-like base, distally tapered into a conspicuous deltoid beak, distal empty portion of 1.2–1.9 mm long. ***Achenes*** 2.1–3.0 × 1.1–1.5 mm, with 1.0–1.8 mm long from base to the widest point, ovate to obovate, flat to plano-convex, the style sessile. ***Rachilla*** smooth, the exserted portion 1.8–3.8 mm long, erect, the hook slightly bend respect to the rachilla axis. ***Anthers*** filament linear, flat, as wide as the anthers.

###### Distribution.

Tristan da Cunha archipelago in the S Atlantic Ocean (Fig. 1), in the islands of Tristan da Cunha, Gough, Inaccessible and Nightingale.

###### Notes.

The specimen selected as the lectotype matches the illustration of tab. VI from “Esquisse Fl. Tristan d’Acugna”.

The etymology *brevicaulis* may seem confusing for a plant up to 61 cm tall. The name was coined by du Petit-Thouars (1808) to refer to short-stemmed plants, as opposed to what he simultaneously called *Carex ramosa* Thouars, nom. illeg. and *Uncinia gracilis* Thouars, nom. illeg., also from Tristan da Cunha, a plant with well-developed stems. Later, in the reprint of “Esquisse de la Flore de Tristan d’Acugna” (du Petit-Thouars 1811), the name was republished as *Carex hamosa*. These three names were obliterated from the literature because they are nomenclaturally superfluous with *C. uncinata* L.f., as this name is cited immediately below as a synonym, despite having priority over them. The plant is represented in tab. V from “Esquisse de la Flore de Tristan d’Acugna”, and it matches the specimen P04023166! [*Uncinia gracilis*, Tristan d’Acugna, Herbier du Petit-Thouars], which is an elongated specimen of *C. brevicaulis* Thouars.

When coining *U. brevicaulis* var. *gracilior*, Hemsley cited in the protologue “*Uncinia gracilis* Thouars [...] (excl. synon.).” Accordingly, he was referring his taxonomic concept of var. *gracilior* to du Petit-Thouars’s *U. gracilis* but excluding the type of *C. uncinata* L.f. This nomenclatural act resulted in a legitimate name.

##### Carex donjuanii

Taxon classificationPlantaePoalesCyperaceae

García-Moro, Roalson, Rouhan & Jim.Mejías nom. nov.

20FEE260-B5A6-52F2-B129-BFE6CD58BEB8

urn:lsid:ipni.org:names:77387594-1

Figs 5B, 6B

≡ Uncinia rigida Boeckeler in Flora 65: 64 (1882) syn. subs. ≡ Uncinia brevicaulis var. *rigida* (Boeckeler) Kük., Pflanzenr. (Engler) IV, 20(38): 52 (1909). Lectotype (**here designated**). Île St. Paul • 6 Dec 1874, Expédition astronomique aux Iles St-Paul et d’Amsterdam, 1874–75, *G. de l’Isle 7* (P04023174!; isolectotype: P04343662!); syntypes: Île St. Paul • côte Sud d’Ile, 14 Nov 1874, Expédition astronomique aux Iles St-Paul et d’Amsterdam, 1874–75, *G. de l’Isle 7* (K000960342!; P04343674!).
= Uncinia brevicaulis var. *robustior* Hemsl., Rep. Voy. Challenger, Bot. 1(3): 159, 167, tab. XLV (1885). Lectotype (**here designated**). Amsterdam • *G. de l’Isle 44*, 23 Dec 1874 (P04023171!; isolectotype: P04023172!).

###### Etymology.

This species is named after Dr. Juan Antonio Sánchez Rodríguez, retired Associate Professor at the University of Salamanca and a specialist in plants from the Iberian Central System. Dr. Sánchez Rodríguez was respectfully referred to as “Don Juan” (Mr. Juan) by botany students at the University of Salamanca. I (P.G.-M.) name this species in his honour as a tribute to his lifelong dedication and outstanding contribution to both academic research and scientific outreach in Spanish botany. In recognition of his passion for teaching and his remarkable ability to transmit a deep love for the plant world, which profoundly inspired me to embark on a research career and ultimately led me to where I am today.

###### Description.

***Plants*** caespitose, with short rhizomes. ***Fertile culms*** 7.0–51.0 cm long, 0.9–2.0 mm wide, erect to slightly curved, equalling to longer than leaves, subrounded to obtusely trigonous, smooth. ***Leaves*** 6–35 cm long, the widest ones 3.6–5.2 mm wide, the uppermost leaf 2.0–5.2 mm wide, coriaceous, section flat or C- to V-shaped, the margins antrorsely scabrid. ***Inflorescence*** a solitary, androgynous spike, 3.0–9.5 cm × 3.8–8.6 mm, bractless, long-oblong, cylindrical to slightly subclavate, densely flowered, usually with more than 50 pistillate flowers, ripe utricles adpressed to ascendant; staminate portion 4.0–13.0 mm long. ***Pistillate glumes*** from the middle part of the spike 5.6–8.2 × 1.7–2.9 mm, with 2.5–4.4 mm long from the base to the widest point and 1.0–1.7 mm wide half-length from the widest point to the tip, slightly longer to equalling the utricles, elliptic, attenuated into an acute apex, membranaceous to subcoriaceous, surface glabrous, straw-colored with the middle part pale-brownish and narrow hyaline borders, margins sparsely antrorsely scabrid towards the apex, with only the central vein slightly marked or veinless. ***Utricles*** 4.8–6.9 × 1.5–2 mm, with 2.3–3.7 mm long from the base to the widest point, oblong to elliptic, plano-convex, pale to dark brownish, pilose with adpressed hairs on the sides more densely arranged towards the tip, the margins with spreading hairs mostly arranged into dense fascicles, without evident prominent lateral veins, with several slightly raised veins adaxially and abaxially, proximally tapered to a substipitate-like base, distally tapered into an inconspicuous deltoid beak, distal empty portion of 1.2–2.2 mm long. ***Achenes*** 2.7–3.4 × 1.2–1.7 mm, with 1.3–2.1 mm long from base to the widest point, ovate-oblong, biconvex, the style sessile. ***Rachilla*** smooth, the exserted portion 2.4–3.9 mm long, erect, the hook slightly bend respect to the rachilla axis. ***Anthers*** filament linear, slightly flattened towards the apex, the distal part as wide as the anthers.

###### Distribution.

Amsterdam and Saint Paul islands, in the S Indian Ocean.

###### Notes.

The combination *Carex rigida* is already occupied (*Carex rigida* Schrank = *Carex ferruginea* Scop.), and the new name *C. donjuanii* is therefore proposed here.

G. de l’Isle’s numbers do not seem to be collection numbers but rather some sort of taxon numbering. In the original material of *Uncinia rigida* cited above, the number G. de l’Isle 7 corresponds to two different collection dates: 6 December 1874 and 14 November 1874. Since the collection from 6 December is lectotypified here, the one from 14 November is to be considered a syntype.

##### Carex haleakalensis

Taxon classificationPlantaePoalesCyperaceae

Jim.Mejías, Larridon, Roalson & García-Moro sp. nov.

19A01C4D-1851-5C06-AADB-A17E8385A9D0

urn:lsid:ipni.org:names:77387595-1

Figs 5C, 6C

###### Holotype.

Hawaii • East Maui, Kalapawili ridge above NE end of Haleakala Crater, 19 Jul 1969, *J. Henrickson & R. Vogl 3894* (US3090900! barcode 00436681; isotype: NY03091289!).

###### Diagnosis.

Similar to *C. delacosta* Kunze, from which it can be distinguished by its smaller utricles and spikes (see identification key).

###### Etymology.

This species is named after the geographic area where it is found—the grass-dominated ridges around and radiating from the rim of Haleakalā Crater at the summit of East Maui.

###### Description.

***Plants*** caespitose, with short rhizomes. ***Fertile culms*** 12–43 cm long, 0.7–1.1 mm wide, erect to slightly curved, shorter to longer than leaves, rarely flat to acutely trigonous, smooth. ***Leaves*** 10–26 cm long, the widest ones 2.3–4.0 mm wide, the uppermost leaf 1.5–2.7 mm wide, coriaceous to subcoriaceous, section flat to C-shaped, occasionally slightly M-shaped, the margins antrorsely scabrid. ***Inflorescence*** a solitary, androgynous spike, 3.0–5.6 cm × 2.5–3.0 mm, bractless or occasionally the lowermost glume developing into a setaceous bract up to half-length of the spike, never surpassing it, long-oblong to linear, densely flowered, with ca. 20–30 pistillate flowers, ripe utricles adpressed to ascendant; staminate portion 3.6–10.0 mm long. ***Pistillate glumes*** from the middle part of the spike 4.0–4.8 × 1.8–2.5 mm, with 2.0–2.8 mm from base to the widest point, 1.2–2.0 mm wide at half-length from the widest point to the tip, shorter to equalling the utricles, elliptic to ovate, attenuated into an obtuse apex, membranaceous, surface glabrous, straw-colored with the central part paler, narrowly brownish towards the sides and also narrow hyaline borders, margins densely and irregularly antrorsely scabrid, one prominent central vein, usually greenish or straw-colored, the sides sometimes faintly several veined. ***Utricles*** 4.0–4.4 × 1.2–1.5 mm, with 1.8–2.8 mm long from base to its widest point, elliptic, flat to plano-convex, covered reddish-brownish linear spots, usually pale yellowish proximally, pilose with adpressed hairs on sides and both adaxially and abaxially, more densely arranged towards the apex, without prominent lateral veins, without evident veins adaxially or abaxially, proximally tapered to a substipitate-like base, distally tapered into an inconspicuous deltoid beak, distal empty portion of 0.7–1.0 mm long. ***Achenes*** 2.2–2.6 × 1.0–1.3 mm, with 0.7–2.2 mm long from base to the widest point, obovate-oblong, flat to plano-convex, the style slightly elevated on a neck-like projection of the achene. ***Rachilla*** smooth, the exserted portion 2.0–3.0 mm long, erect, the hook slightly bend respect to the rachilla axis. ***Anthers*** filament linear, flat, anthers not observed.

###### Distribution.

Hawaii, only known from the island of Maui.

###### Additional material studied (paratypes).

**Hawaii** • Maui, Kipahulu valley, National Park lands, Puu Alaea region and ridge up to summit of Pohaku and Lauulu, 26 Sep 1997, *K. R. Wood, S. Perlman, P. Weltonm & W. Haus 6778*, (US3584238! barcode 01076792; NY03091281!); • Kaleanae [illegible], 03 Aug 1919, *C. N. Forbes 1047m* (US3509393! barcode 01052822); • East Maui, Hana District, Upper Hana Forest Reserve, 14 Aug 1973, *B. Harrison 438* (BISH 1011028!).

#### *Carex austrocompacta* group

Measurements of key characters may overlap in some couplets. The identification of specimens should be fitted to the best matching couplet.

**Table:** 

1	Spikes 5.9–8.8 mm wide; widest leaves up to 2.4–4.5 mm; utricles width up to 1.4–1.9 mm; pistillate glumes 6.1–8.7 mm long; plants from Marion-Prince Edward and Crozet archipelagos	***C. dikei***
–	Without the precedent combination of characters	**2**
2	Plants with low growth habit, the fertile culms 5–13 cm long; utricle width 1.0–1.4 mm; spikes 1.5–2.2 cm long	**3**
–	Without the precedent combination of characters	**4**
3	Utricles 1.0–1.4 mm wide, with an empty distal portion 1.4–2.0 mm long; fertile culms 0.7–1 mm wide; the hook of the rachilla 1.2–1.8 mm long; plants from Kerguelen archipelago	***C. moseleyana***
–	Utricles 0.9–1.1 mm wide, with an empty distal portion 1.1–1.5 mm; fertile culms 0.5–0.6 mm wide at the middle part; hook of the rachilla 1.8–2.1 mm long; plants from the Pacific SW subantarctic archipelagos	***C. erebus***
4	Utricles 1.8–2.0 mm wide; achene ca. 2 mm wide; plants from SE Australia and Tasmania	***C. austrocompacta***
–	Utricles 0.7–1.4 mm wide; achene 0.6–1.4 mm wide	**5**
5	***C. nao-victoriae***
–	The (1)2–3(4) proximal-most glumes generally well-developed into setaceous bracts that exceeds the inflorescence length; utricles 3.2–4.9 mm long, rarely longer; exserted part of the rachilla typically 3.2–4.9(5.1) mm long; leaves 6.5–20(22) cm long, shorter to equalling the flowering stems; number of pistillate flowers ca. 15–20; plants from Tristan da Cunha archipelago	***C. moro-ortegae***

##### Carex dikei

Taxon classificationPlantaePoalesCyperaceae

(Nelmes) K.L.Wilson, Bot. J. Linn. Soc. 179: 31 (2015).

365CD2E5-6E10-5114-B177-71CB5F692BEF

Figs 5D, 6D

≡ Uncinia dikei Nelmes [dykei], Kew Bull. 4(3): 377 (1949) [basionym]

###### Holotype.

Marion Island • *Mr. Dike [Dyke] s.n*., Feb.-March 1948 (K000960343!)

###### Etymology.

Named after the collector of the holotype, Mr. Dike, who originally spelt himself as “Dyke.”

###### Distribution.

Marion and Prince Edward Islands and Crozet archipelago at the Île de la Possession, Île de l’Est, and Île aux Cochons, in the S Indian Ocean.

###### Description.

***Plants*** laxly caespitose, with short- to long-elongated rhizomes. ***Fertile culms*** 5.5–24 cm long, 0.9–1.4 mm wide, erect to slightly curved, shorter to longer than leaves, acutely trigonous, smooth. ***Leaves*** 6–19 cm long, the widest ones 2.8–4.5 mm wide, the uppermost leaf (1.4)1.7–2.9 mm wide, coriaceous to subcoriaceous, C-shaped to V-shaped, sometimes slightly M-shaped, the margins antrorsely scabrid. ***Inflorescence*** a solitary, androgynous spike, 2.1–3.5 cm × 5.9–8.8 mm, the lowermost glume developing into a setaceous bract usually longer than the spike, narrowly obovate to obovate-elliptic or subclavate, densely flowered, with ca. 30 pistillate flowers, ripe utricles ascendant; staminate portion 4.1–6.7 mm long. ***Pistillate glumes*** from the middle part of the spike 6.15–8.5(8.7) × 2.–2.8 mm, with 1–1.7 mm from base to the widest point, 1.2–1.6 mm wide at half-length from the widest point to the tip, longer than the utricles, narrowly ovate, the apex acute, membranaceous, glabrous, narrowly straw-colored in the central part, the sides browni to orangish-brown, sometimes proximal portion darker than the distal portion, margins narrowly hyaline, glabrous, 1–3 prominent central raised veins, paler, sometimes straw-colored, lateral veins faint, mostly noticeable at the glume base, not extending distally. ***Utricles*** 4.4–6.1 × 1.4–1.9 mm, with 2–3.3 mm long from base to its widest point, 0.9–1.7 mm wide at half-length from the widest point to the tip, elliptic to fusiform, sometimes narrowly ovate, flat to plano-convex, sometimes subtrigonous, greenish to straw-colored, distal portion brownish, glabrous, two prominent lateral veins, with few more or less conspicuous veins adaxially and several raised veins abaxially, proximally abruptly attenuated to a stipitate-like base with 0.5–1.3 mm long and 0.3–0.7 mm wide, distally tapered into a conical conspicuous beak, distal empty portion of 1–2.1 mm long. ***Achenes*** 2.1–3.1 × 1–1.5 mm, with 1.1–1.5 mm long from base to the widest point, 0.9–1.3 mm wide at half-length from the widest point to the tip, oblong, trigonous, the style elevated on a short flattened appendage. ***Rachilla*** smooth, the exserted portion (3.8)4–5.5 mm long, erect, the hook 1.2–2.1 mm long, slightly bend respect to the rachilla axis. ***Anthers*** filament filiform, narrower than the anthers.

###### Distribution.

Marion and Prince Edward islands, and Crozet Archipelago (known from Île de la Possession, Île aux Cochons, and Île de l’Est), in the S Indian Ocean.

###### Notes.

Although material from the Prince Edward system was not studied, the citation of *Uncinia compacta* there by Gremmen (1981) is considered reliable and surely refers to what is here considered *C. dikei*.

##### Carex erebus

Taxon classificationPlantaePoalesCyperaceae

K.A.Ford, Bot. J. Linn. Soc. 179: 31 (2015).

2F3A1B48-F44D-5A58-B76C-3BE5A6AD615F

Figs 5E, 6E

≡ Uncinia hookeri Boott, in Hook. f. Fl. Antarct.: 91 (1844). t. 51, repl. Syn. ≡ Uncinia riparia var. hookeri (Boott) Kük., Pflanzenr. (Engler) 38, 63 (1909). Lectotype (**here designated**). Lord Auckland Island • Hooker’s voyage (K000357213!); syntypes: Campbell Island • 1840 (K000357214!); Lord Auckland Islands • 1840 (K000357216 digital image!); • Antart. Exp. 1839–1843, JDH (E00386922 digital image!).

###### Etymology.

Named for the ship HMS Erebus on which Joseph Dalton Hooker sailed on the Voyage to the Antarctic 1839–1843, during which this species was first collected from the Auckland Islands (K.A.Ford in Global Carex Group, 2015).

###### Description.

***Plants*** laxly to densely matted, with short-elongated rhizomes. ***Fertile culms*** 5–7 cm long, ca. 0.5 mm wide, erect to slightly curved, generally shorter than leaves, rarely equalling them, flat to obtusely trigonous, smooth. ***Leaves*** 7.5–17.0 cm long, the widest ones 1.9–2.4 mm wide, the uppermost leaf 1.5–2 mm wide, subcoriaceous, C-shaped to slightly V-shaped, the margins antrorsely scabrid, rarely smooth, commonly bearing simple trichomes, but also with compound trichomes formed by two or more teeth, occasionally also with hook-shaped antrorse trichomes. ***Inflorescence*** a solitary, androgynous spike, 1.6–2.2 cm × 3.2–5.0 mm, the lowermost glume developing into a setaceous bract usually longer than the spike, elliptic to obovate or subclavate, laxly to densely flowered, with ca. 10–30 pistillate flowers, ripe utricles ascendant; staminate portion 6.2–8.5 mm long. ***Pistillate glumes*** from the middle part of the spike 4.7–5.5 × 1.4–1.9 mm, with 1–1.6 mm from base to the widest point, 0.9–1.5 mm wide at half-length from the widest point to the tip, longer to equalling the utricles, narrowly ovate to elliptic, the apex acute, membranaceous, glabrous, greenish-hyaline to brownish, margins sometimes narrowly hyaline, with 1–3 prominent central raised veins, greenish to dark-brownish, with some faint veins proximally, usually not reaching half-length of the glume. ***Utricles*** 4–5 × 0.9–1.1 mm, with 1.7–2.2 mm long from base to its widest point, ca. 0.65 mm wide at half-length from the widest point to the tip, narrowly ovate, elliptic to fusiform, trigonous to subtrigonous, sometimes plano-convex, greenish to dark brown, glabrous, with two prominent lateral veins, more or less conspicuously veined adaxially, with few to several raised veins abaxially, proximally abruptly attenuated to a stipitate-like base with 0.1–1 mm long and 0.1–0.3 mm wide, distally tapered into a conical conspicuous beak, distal empty portion of 1.1–1.5 mm long. ***Achenes*** 1.9–2.5 × 0.8–0.9 mm, with 1–1.3 mm long from base to the widest point, ca. 0.75 mm wide at half-length from the widest point to the tip, oblong to elliptic, trigonous to subtrigonous, the style elevated on a short flattened appendage. ***Rachilla*** smooth, the exserted portion 4–4.8 mm long, erect, the hook 1.8–2.1 mm long, slightly bend respect to the rachilla axis. ***Anthers*** filament filiform, narrower than the anthers. [Description completed with Moore and Edgar 1970].

###### Distribution.

Macquarie, Auckland, Campbell, Stewart, and Antipodes islands, in the SW Pacific.

###### Notes.

Plants vary from lax to dense mats according to the habitat, which ranges from shrubland, tussock grassland, herbfield, and fellfield to occasionally the understory of *Metrosideros* and *Dracophyllum* forest. Lehnebach (2010) indicated a type for *Uncinia hookeri* Boott. However, he did not use wording that can be considered a formal lectotypification. Accordingly, the lectotype is designated here on the same specimen he mentioned.

##### Carex moro-ortegae

Taxon classificationPlantaePoalesCyperaceae

García-Moro & Jim.Mejías sp. nov.

26B36DC8-3DC1-5004-A937-9C61679379D5

urn:lsid:ipni.org:names:77387596-1

Figs 5F, 6F

###### Holotype.

Tristan da Cunha • 29 Jan 1938, *Y. Mejland 1654* (K000298924!).

###### Diagnosis.

Similar to *C. austrocompacta* K.L.Wilson, from which it can be distinguished by its smaller size of utricles and achenes (see identification key).

###### Etymology.

This species is named after my (P.G.-M.) mother, María Moro Ortega, the kindest person I have ever known, who lovingly cared for me for eighteen beautiful years and who sadly passed away too soon. She lived entirely for her family and friends, caring for everyone around her and always placing others before herself, especially her children. Nearly eleven years later, I am certain that she continues to watch over me from Heaven, and my greatest hope is that she would be proud of the person I have become.

In addition, by the mention to my mother’s surnames, I also wish to indirectly honour other two people. First my grandmother, Pilar Ortega, who was in many ways a second mother to me, having lived with us throughout those same eighteen years. One of the strongest people I have ever known, she faced the early loss of her husband while raising two daughters on her own, and later endured the loss of one of them, yet never ceased to fight for her family. And second, my aunt María del Pilar Moro Ortega, who, in the absence of my mother and grandmother, was always kind to me and encouraged me in pursuing my goals. She welcomed me into her home and cared for me as her son. She helped me through my most difficult moments and never lost faith in me, even when I had already lost it myself.

To these three women I owe some of the most important lessons of my life: unconditional love for those we cherish, devotion to family, selfless commitment, and a boundless capacity to give everything for the people we love. As a symbolic reflection of this, the spikes of the newly described C. moro-ortegae are often accompanied by three leaf-like bracts, echoing my mother, my aunt, and my grandmother.

###### Description.

***Plants*** laxly caespitose, with short-elongated rhizomes. ***Fertile culms*** 9.5–25 cm long 0.5–1.3 mm wide, erect to slightly curved, longer than leaves, trigonous, smooth. ***Leaves*** 6.5–22 cm long, the widest ones 1.5–3 mm wide, the uppermost leaf 1–2.4 mm wide, subcoriaceous to coriaceous, C-shape, sometimes V-shaped, the margins antrorsely scabrid. ***Inflorescence*** a solitary, androgynous spike, 1.7–4.8 cm × 2.3–5 mm, the (1)2–3(4) proximal-most glumes generally well-developed into setaceous bracts that exceeds the inflorescence length, long-oblong, sometimes narrowly obovate, subclavate to linear, densely flowered, with less than 15–20 pistillate flowers, ripe utricles ascendant; staminate portion 2.8–9 mm long. ***Pistillate glumes*** from the middle part of the spike 4–7 × 1.3–2.2 mm, with 0.3–2(2.5) mm from base to the widest point, 0.5–1.5 mm wide at half-length from the widest point to the tip, longer than the utricles, narrowly deltoid to ovate, the apex acute, herbaceous, glabrous, mostly hyaline, greenish to straw-colored, becoming brownish towards the borders, more rarely entirely pale-brownish, with narrowly hyaline margins, glabrous, 1–3 prominent central slightly-raised veins, paler than the glume, lateral veins faint restricted to the base. ***Utricles*** 3.2–4.9 × 0.7–1.4 mm, with 1.5–2.8 mm long from base to its widest point, 0.5–0.9 mm wide at half-length from the widest point to the tip, elliptic, subtrigonous, sometimes plano-convex, greenish to yellowish, sometimes brownish, sometimes whitish, glabrous, two prominent lateral veins, few faintly veined adaxially, sometimes inconspicous, with few slightly-raised veins to sometimes faintly veined abaxially, proximally abruptly attenuated to a stipitate-like base with 0.4–1.1 mm long and 0.2–0.4 mm wide, distally tapered into a conical conspicuous beak, distal empty portion of 0.9–1.6 mm long. ***Achenes*** 1.3–2.5 × 0.6–1.2 mm, with 0.5–1.9 mm long from base to the widest point, 0.5–1.1 mm wide at half-length from the widest point to the tip, oblong to elliptic, sometimes obovate, subrounded, the style sessile. ***Rachilla*** smooth, the exserted portion 3.1–5.1 mm long, erect, the hook 1.2–1.9 mm long, slightly bend respect to the rachilla axis. ***Anthers*** filament filiform, narrower than the anthers.

###### Distribution.

Tristan da Cunha archipelago in the S Atlantic Ocean, known for the islands of Tristan da Cunha, Gough, Inaccessible. Not known for the island of Nightingale.

###### Additional material studied (paratypes).

Tristan da Cunha • Gough Island, 19 Jan 1956, *N.M.Wace 95* (BM000574359!); • 25 Jan 1956, *N.M.Wace 113* (BM000574360!); • 01 Feb 2017, *P.Lambdon PL#062.GI* (K000863169!); • Gony Dale, 03 Jan 2010, *K.Rexer-Huber KRH 18* (K000818138!); • 29 Nov 2016, *P.Lambdon PL#034.GI* (K000863196!); • 09 Dec 2016, *P.Lambdon PL#042.GI* (K000863188!); • Inaccessible Island, 27 Nov 1982, *N.H.Hall 184* (BM000574336!); • 23 Feb 1938, *E.Christophersen 2477* (K000298934!); • 28 Jan 1990, *Ryan 82* (K!); • 19 Feb 1938, *E.Christophersen 2387* (K000298933!); • Tristan Island, Burntwood area, Burntwood, Dale’s Hill, 3 June 2009, *R.L.Halbertsma Gremmen-T07 0114* (K000649074!); • 28 Jan 1938, *Y.Mejland 1379* (K000298918!); • 28 Jan 1938, *E.Y.Mejland 1371* (K000298926!); • 13 Jan 1938, *E.Christophersen & Y.Mejland 1065* (K000298921!); • 02 Dec 1976, *N.M.Wace T32*0 (K000298932!); • 19 Feb 1938, *Y.Mejland 1517* (K000298919!); • 07 Jan 1938, *E.Christophersen & Y.Mejland 516* (K000298920!); • 28 Jan 1938, *Y.Mejland 1368* (K000298929!); • 21 Dec 1937, *E.Christophersen & Y.Mejland 64* (K000298922!); • 13 Apr 1963, *N.M.Wace T228* (K000298930!); • 28 Jan 1938, *Y.Mejland 1654* (K000298924!); • 13 Mar 1968, *N.M.Wace T168* (K000298931!); • 21 Dec 1937, *E.Christophersen & Y.Mejland 43* (K000298927!); • 28 Jan 1938, *Y.Mejland 1387* (K000298923!).

###### Notes.

The studied populations of *C. moro-ortegae* exhibited great variability in morphology and habitat (bogs, crevices, heathlands, and slopes). The observed morphological variation could be due to ecological differences, but the fragmentary information provided on the herbarium vouchers prevents the establishment of a relationship between habitat and morphology. Alternatively, it cannot be entirely discarded that these differences were the result of *in situ* diversification and that more than one taxon might be recognized in Tristan da Cunha. Future studies may help elucidate the situation in this archipelago.

##### Carex moseleyana

Taxon classificationPlantaePoalesCyperaceae

(Boeckeler) Jim.Mejías & García-Moro comb. nov.

FD233C29-6C28-55F7-BC34-377EECD28BD7

urn:lsid:ipni.org:names:77387597-1

Figs 5G, 6G

≡ Uncinia moseleyana Boeckeler, Flora 61: 170 (1878) [basionym]. Lectotype (**here designated**). Kerguelen • [illegible], Challenger Expedition, *Moseley s.n*., (BM000574364 digital image!; isolectotypes: P04023167!; S-G-9578 digital image!).

###### Etymology.

Named after H.N.Moseley, the British 19^th^-century botanist who took part in the Challenger expedition, collector of the original material.

###### Description.

***Plants*** laxly caespitose, with short-elongated rhizomes. ***Fertile culms*** 5–13 cm long, 0.7–1 mm wide, erect to slightly curved, shorter to equalling the leaves, acutely trigonous, smooth. ***Leaves*** 2–11 cm long, the widest ones 2.2–2.5 mm wide, the uppermost leaf 1.4–3.5 mm wide, coriaceous to subcoriaceous, flat to C-shaped, sometimes V-shaped, the margins antrorsely scabrid. ***Inflorescence*** a solitary, androgynous spike, 1.5–2.1 cm × 4.2–5.5 mm, bractless or occasionally the lowermost glume developing into a setaceous bract up to as long as the spike, elliptic to fusiform, densely flowered, with ca. 20 pistillate flowers, ripe utricles ascendant; staminate portion 3.2–9.7 mm long. ***Pistillate glumes*** from the middle part of the spike 4.5–6.6 × 1.7–2.1 mm, with 0.8–2.3 mm from base to the widest point, 1–1.6 mm wide at half-length from the widest point to the tip, longer than the utricles, narrowly ovate, the apex acute, membranaceous, surface glabrous, the central part narrowly straw-colored, the sides dark brown, especially at the distal portion, the margins somewhat paler, glabrous, 1–3 prominent central raised veins, straw-colored, and several lateral veins most noticeable at the base and some reaching the middle part of the glume. ***Utricles*** 4.5–5.2 × 1–1.4 mm, with 2.3–2.8 mm long from base to its widest point, 0.7–1 mm wide at half-length from the widest point to the tip, ovate to elliptic, subtrigonous, straw-colored to yellowish, distal portion brownish, glabrous, two prominent lateral veins, with few faint veins adaxially and several raised conspicuous veins abaxially, proximally abruptly attenuated to a stipitate-like base with 0.7–1.6 mm long and 0.3–0.8 mm wide, distally tapered into a conical conspicuous beak, distal empty portion of 1.4–2 mm long. ***Achenes*** 1.7–2.4 × 0.5–1.2 mm, with 0.9–1.3 mm long from base to the widest point, 0.6–1.1 mm wide at half-length from the widest point to the tip, oblong, trigonous, the style elevated on a short flattened appendage. ***Rachilla*** smooth, the exserted portion 3.6–4.7 mm long, erect, yellowish, the hook 1.2–1.8 mm long, reddish-tinged, sulcate on the sides, slightly bend respect to the rachilla axis. ***Anthers*** filament filiform, narrower than the anthers.

###### Distribution.

Kerguelen archipelago, in the S Indian Ocean.

##### Carex nao-victoriae

Taxon classificationPlantaePoalesCyperaceae

García-Moro, Rouhan & Jim.Mejías sp. nov.

EFA6BEFC-F620-50DC-BDB1-E5BD7C609ED8

urn:lsid:ipni.org:names:77387598-1

Figs 5H, 6H

###### Holotype.

Nouvelle Amsterdam • Pres de la forêt de *Phylica*. 11 Dec 1963, *A. Lourteig & P. Cour 41* (P04343692!; isotypes: P04343690!, P04343692!, P04343695!).

###### Diagnosis.

Similar to *C. austrocompacta* K.L.Wilson, from which it can be distinguished by its smaller utricles and spikes (see identification key).

###### Etymology.

The name refers to the *Nao Victoria*, the only ship from the first circumnavigation expedition that completed the trip around the world, first under the command of Fernando Magallanes from Spain to the Philippines and later under the command of Juan Sebastián Elcano from there back to Spain. Amsterdam Island was first reported by a human from this ship. The name was chosen to commemorate the historical feat that the first circumnavigation of the world represented for humanity. The name is intended to continue the dedication of names to historical ships in *Carex*, started by B.G. Hamlin with *C. elingamita* Hamlin, followed by *C. erebus* K.A.Ford, and now continued here.

= *Uncinia compacta* var. *elongata* C.B.Clarke in J. Linn. Soc., Bot. 20: 395 (1883). ≡ *Uncinia hookeri* var. *elongata* (C.B.Clarke) Hamlin in Trans. Roy. Soc. New Zealand, Bot. 10(2): 130 (1963). Lectotype (**here designated**). Amsterdam • Expédition astronomique aux Iles St-Paul et d’Amsterdam, 1874–75, *G. de l’Isle 55* (P04023170!); syntype: *G. de l’Isle 55* (P04023169!).

###### Description.

***Plants*** laxly caespitose, with short-elongated ascending rhizomes. ***Fertile culms*** 10–17 cm long, 0.6–0.8 mm wide, erect to slightly curved, usually shorter than leaves rarely slightly surpassing them, sometimes concealed by the leaves, acutely trigonous, smooth. ***Leaves*** 12–38 cm long, the widest ones 2–3 mm wide, the uppermost leaf 0.9–1.7 mm wide, subcoriaceous to herbaceous, flat to V-shaped, sometimes slightly C-shaped, the margins antrorsely scabrid. ***Inflorescence*** a solitary, androgynous spike, 2.4–3.4 cm × 4.2–5.6 mm, bractless or occasionally the lowermost glume developing into a setaceous bract up to as long as the spike, never surpassing it, long-elliptic to long-obovoid or subclavate, densely flowered, with ca. 30 pistillate flowers, ripe utricles ascendant, those from the proximal portion slightly erect; staminate portion 5.5–7.5 mm long. ***Pistillate glumes*** from the middle part of the spike 5.2–5.5 × 1.5–2.2 mm, with 0.8–1.9 mm from base to the widest point, 1.1–1.3 mm wide at half-length from the widest point to the tip, longer to equalling the utricles, long-elliptic to narrowly ovate, the apex obtuse, membranaceous, surface glabrous, straw-colored, brownish towards the borders, especially towards the distal margins, glabrous, (2)3 prominent central raised veins, light-brownish, the sides sometimes faintly several veined proximally. ***Utricles*** (4.7)4.9–5.3 × 1.1–1.4 mm, with 2.0–2.7 mm long from base to its widest point, 0.7–1 mm wide at half-length from the widest point to the tip, ovate to narrowly-ovate, subtrigonous to plano-convex, straw-colored to greenish, glabrous, two prominent lateral veins, with several faint veins adaxially and several raised conspicuous veins abaxially, proximally abruptly attenuated to a stipitate-like base with 0.7–1.7 mm long and 0.3–0.5 mm wide, distally tapered into a conical conspicuous beak, distal empty portion of 1.2–1.5 mm long. ***Achenes*** 2.3–2.8 × 1.1–1.4 mm, with 0.9–1.3 mm long from base to the widest point, 0.8–1.1 mm wide at half-length from the widest point to the tip, oblong, trigonous to plano-convex, the style sessile. ***Rachilla*** smooth, yellowish, the exserted portion 5.1–6.3 mm long, erect, the hook 1.2–2.1 mm long, yellowish to reddish-tinged, the sides flattened, slightly bend respect to the rachilla axis. ***Anthers*** filament filiform, narrower than the anthers.

###### Distribution.

Amsterdam and Saint Paul islands, in the S Indian Ocean.

###### Additional material studied (paratypes).

Nouvelle Amsterdam • 14 Jan 1985, *J.C.Jolinon 967* (P04343689!); • 30 Dec 1984, *J.C.Jolinon 905* (P04343684!); • 11 Dec 1963, *A.Lourteig & P.Cour 26* (P04343685!).

###### Notes.

G. de l’Isle’s numbers do not unambiguously refer to collections but seem to refer to taxon numbering (see comment for the name *U. rigida* under *C. donjuanii*). Accordingly, the possible duplicate of the lectotype of *U. hookeri* var. *elongata* is regarded here as a syntype.

## Conclusion

This study demonstrates that significant, previously overlooked taxonomic diversity exists within the *Carex brevicaulis* and *C. austrocompacta* complexes across the Pacific and subantarctic archipelagos. Through a standardized morphometric framework, clear morphological discontinuities were identified that support the recognition of six previously unrecognized species. The long-standing underestimation of diversity in these insular groups appears to result from limited inter-archipelago comparisons, fragmented herbarium representation, logistical constraints in remote territories, and the inherent complexity of *Carex* sect. *Uncinia*. These factors have historically promoted broadly circumscribed species concepts. The recognition of four species within the *C. brevicaulis* complex and five within the *C. austrocompacta* complex reflects consistent and diagnosable morphological differentiation across archipelagos. Although some morphogroups show partial overlap in exploratory multivariate analyses, their overall morphological coherence and geographic segregation support their treatment as distinct species. These results underscore the importance of comprehensive, cross-regional taxonomic assessments in remote island systems and highlight the role of integrative approaches in revealing hidden plant diversity. The revised taxonomic treatment presented below provides a framework for accurate identification and future evolutionary and biogeographic studies of these subantarctic and oceanic lineages.

## Supplementary Material

XML Treatment for Carex brevicaulis

XML Treatment for Carex donjuanii

XML Treatment for Carex haleakalensis

XML Treatment for Carex dikei

XML Treatment for Carex erebus

XML Treatment for Carex moro-ortegae

XML Treatment for Carex moseleyana

XML Treatment for Carex nao-victoriae

## Additional information

### Artificial Intelligence (AI) use

Regarding the use of AI in the preparation of this manuscript, the authors declare the following:

ChatGPT (OpenAI, version GPT-5) was used to assist in formatting the references according to the journal’s guidelines.

### Author ORCIDs

P. García-Moro https://orcid.org/0000-0002-8331-4771

R. Lois https://orcid.org/0000-0002-7372-5091

K.A. Ford https://orcid.org/0000-0002-3832-9835

I. Larridon https://orcid.org/0000-0003-0285-722X

G. Rouhan https://orcid.org/0000-0002-0751-5352

E.H. Roalson https://orcid.org/0000-0003-1655-3681

P. Jiménez-Mejías https://orcid.org/0000-0003-2815-4477

### Data availability

All of the data that support the findings of this study are available in the main text or Supplementary Information.

## Supplementary materials

Supplementary material 1 Boxplots of the all the variables measured in *Carex brevicaulis* complex Data type pdf https://binary.pensoft.net/file/1720979This dataset is made available under the Open Database License (http://opendatacommons.org/licenses/odbl/1.0/). The Open Database License (ODbL) is a license agreement intended to allow users to freely share, modify, and use this Dataset while maintaining this same freedom for others, provided that the original source and author(s) are credited. Pablo García-Moro, Raúl Lois, Kerry A. Ford, Isabel Larridon, Germinal Rouhan, Eric H. Roalson, Pedro Jiménez-Mejías

Supplementary material 2 Boxplots of all the variables measured in *Carex austrocompacta* complex Data type pdf https://binary.pensoft.net/file/1720995This dataset is made available under the Open Database License (http://opendatacommons.org/licenses/odbl/1.0/). The Open Database License (ODbL) is a license agreement intended to allow users to freely share, modify, and use this Dataset while maintaining this same freedom for others, provided that the original source and author(s) are credited. Pablo García-Moro, Raúl Lois, Kerry A. Ford, Isabel Larridon, Germinal Rouhan, Eric H. Roalson, Pedro Jiménez-Mejías

Supplementary material 3 Supplementary tables Data type xlsx Explanation note **table SS1**. List of specimens measured and studied. **table S2**. Principal component (PC) loadings for each PCA performed on both species complexes. Variables contributing most strongly to each principal component are highlighted. **table S3**. Confusion matrices of both training and testing datasets obtained from the linear discriminant analyses (LDAs) for each species complex. **table S4**. Linear discriminant (LD) coefficients for each LDA performed. Variables contributing most strongly to each discriminant axis are highlighted. https://binary.pensoft.net/file/1720980This dataset is made available under the Open Database License (http://opendatacommons.org/licenses/odbl/1.0/). The Open Database License (ODbL) is a license agreement intended to allow users to freely share, modify, and use this Dataset while maintaining this same freedom for others, provided that the original source and author(s) are credited. Pablo García-Moro, Raúl Lois, Kerry A. Ford, Isabel Larridon, Germinal Rouhan, Eric H. Roalson, Pedro Jiménez-Mejías
