# Supplementary material for: Hidden diversity at the edges of maps: morphometrics of Carex sect. Uncinia (Cyperaceae) helps unravel taxonomic diversity in subantarctic and remote archipelagos
Source: PhytoKeys. 2026 Jul 21;277:241–67. doi: 10.3897/phytokeys.277.189029 (PMC13416814; doi:10.3897/phytokeys.277.189029)
Supplement: Supplementary material 1 — Boxplots of the all the variables measured in Carex brevicaulis complex [file phytokeys-277-241_article-189029__-s001.pdf]

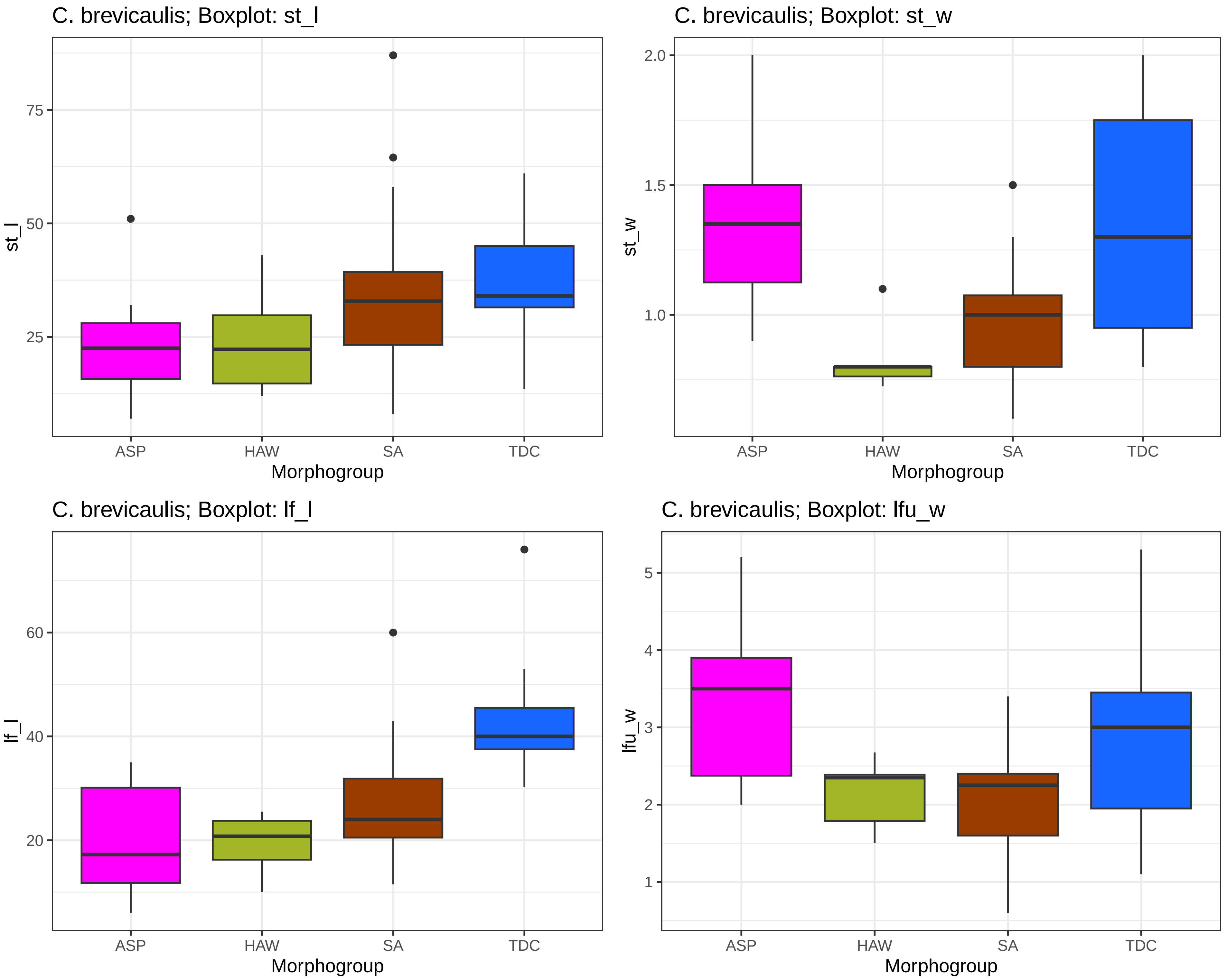

C. brevicaulis; Boxplot: st_l
st_l
25
50
75
ASP
HAW
SA
TDC
Morphogroup
C. brevicaulis; Boxplot: st_w
st_w
1.0
1.5
2.0
ASP
HAW
SA
TDC
Morphogroup
C. brevicaulis; Boxplot: lf_l
lf_l
20
40
60
ASP
HAW
SA
TDC
Morphogroup
C. brevicaulis; Boxplot: lfu_w
lfu_w
1
2
3
4
5
ASP
HAW
SA
TDC
Morphogroup

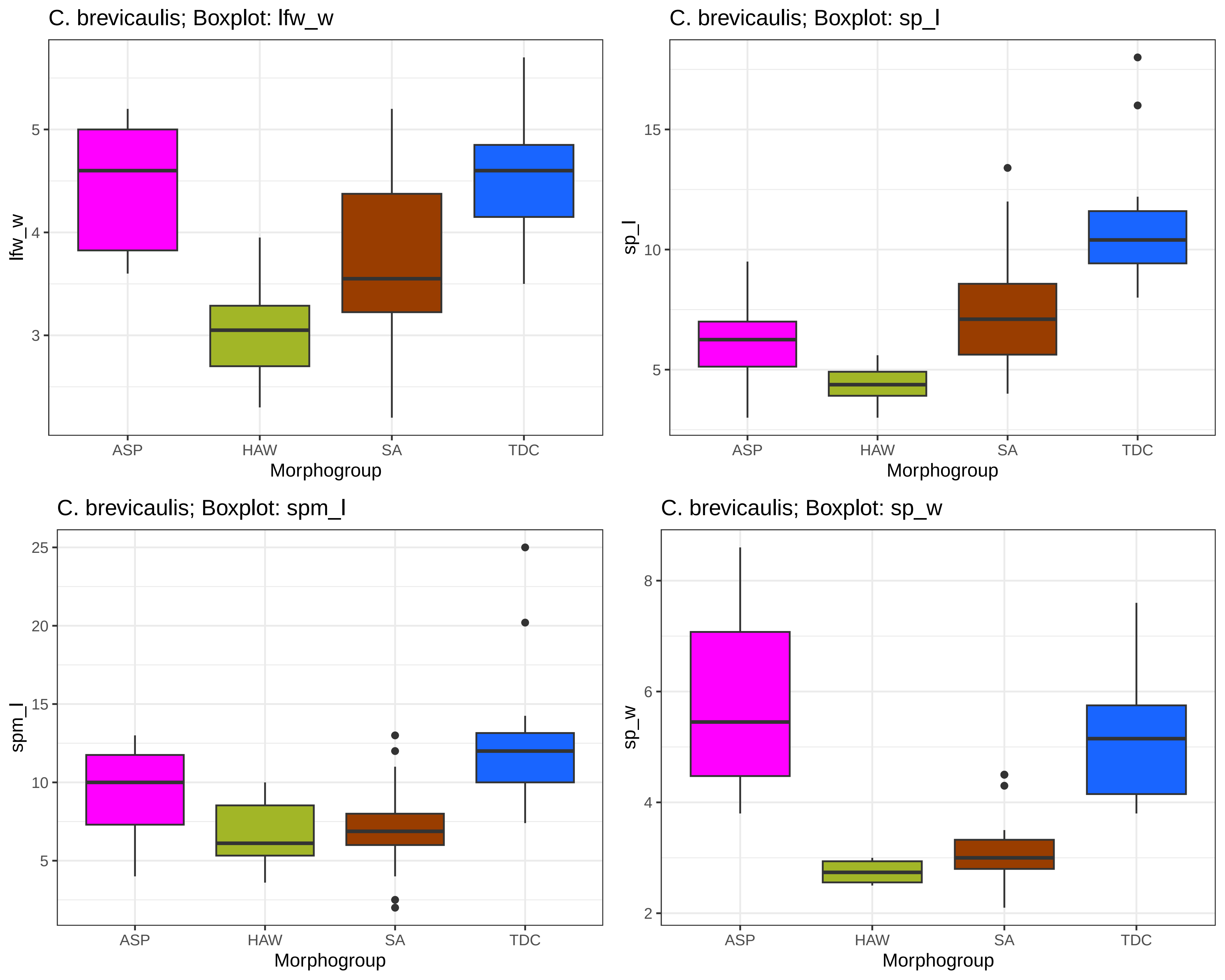
C. brevicaulis; Boxplot: lfw_w
lfw_w
5
4
3
ASP
HAW
SA
TDC
Morphogroup
C. brevicaulis; Boxplot: sp_l
sp_l
15
10
5
ASP
HAW
SA
TDC
Morphogroup
C. brevicaulis; Boxplot: spm_l
spm_l
25
20
15
10
5
ASP
HAW
SA
TDC
Morphogroup
C. brevicaulis; Boxplot: sp_w
sp_w
8
6
4
2
ASP
HAW
SA
TDC
Morphogroup

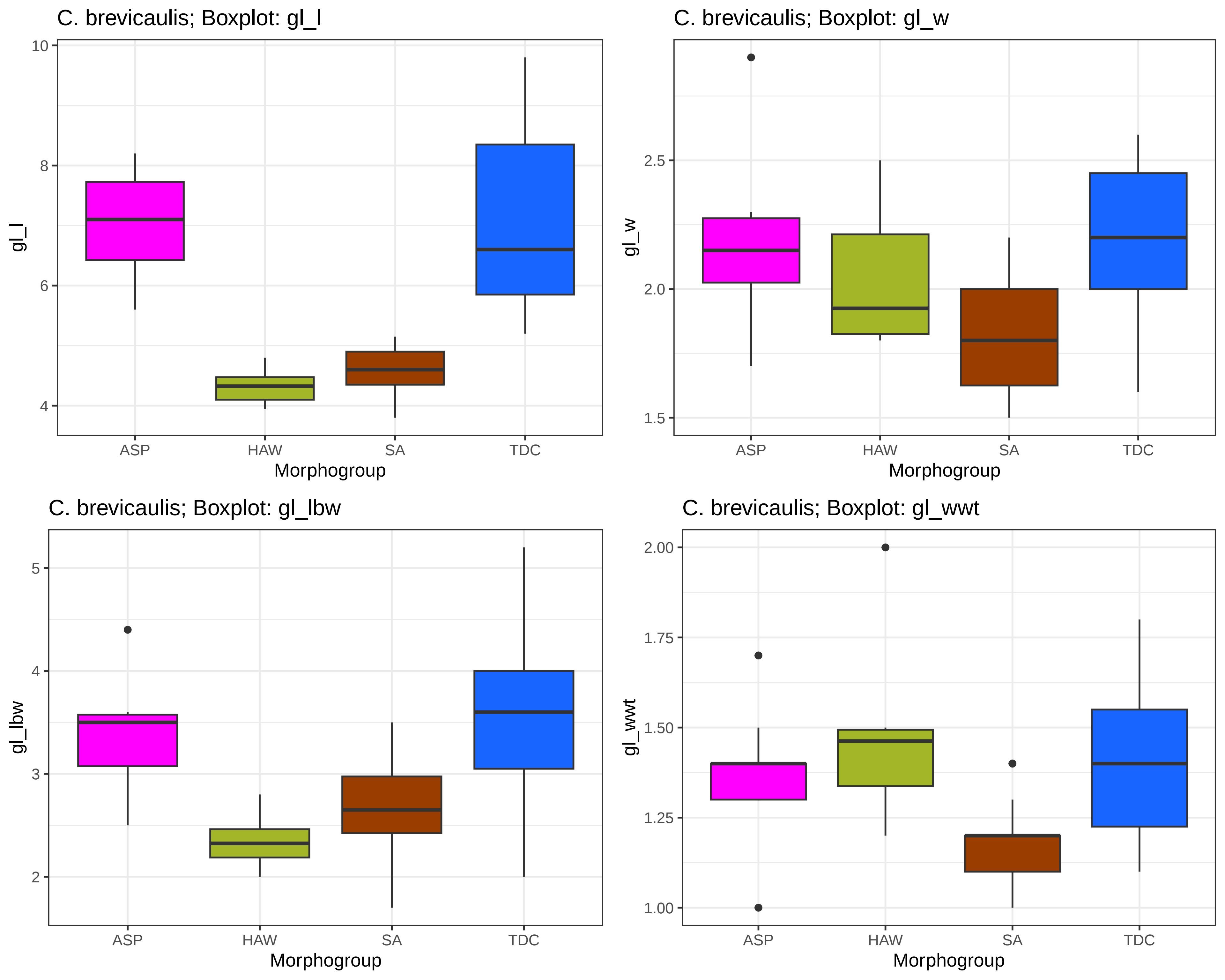
C. brevicaulis; Boxplot: gl_l
gl_l
10
8
6
4
ASP
HAW
SA
TDC
Morphogroup
C. brevicaulis; Boxplot: gl_w
gl_w
2.5
2.0
1.5
ASP
HAW
SA
TDC
Morphogroup
C. brevicaulis; Boxplot: gl_lbw
gl_lbw
5
4
3
2
ASP
HAW
SA
TDC
Morphogroup
C. brevicaulis; Boxplot: gl_wwt
gl_wwt
2.00
1.75
1.50
1.25
1.00
ASP
HAW
SA
TDC
Morphogroup

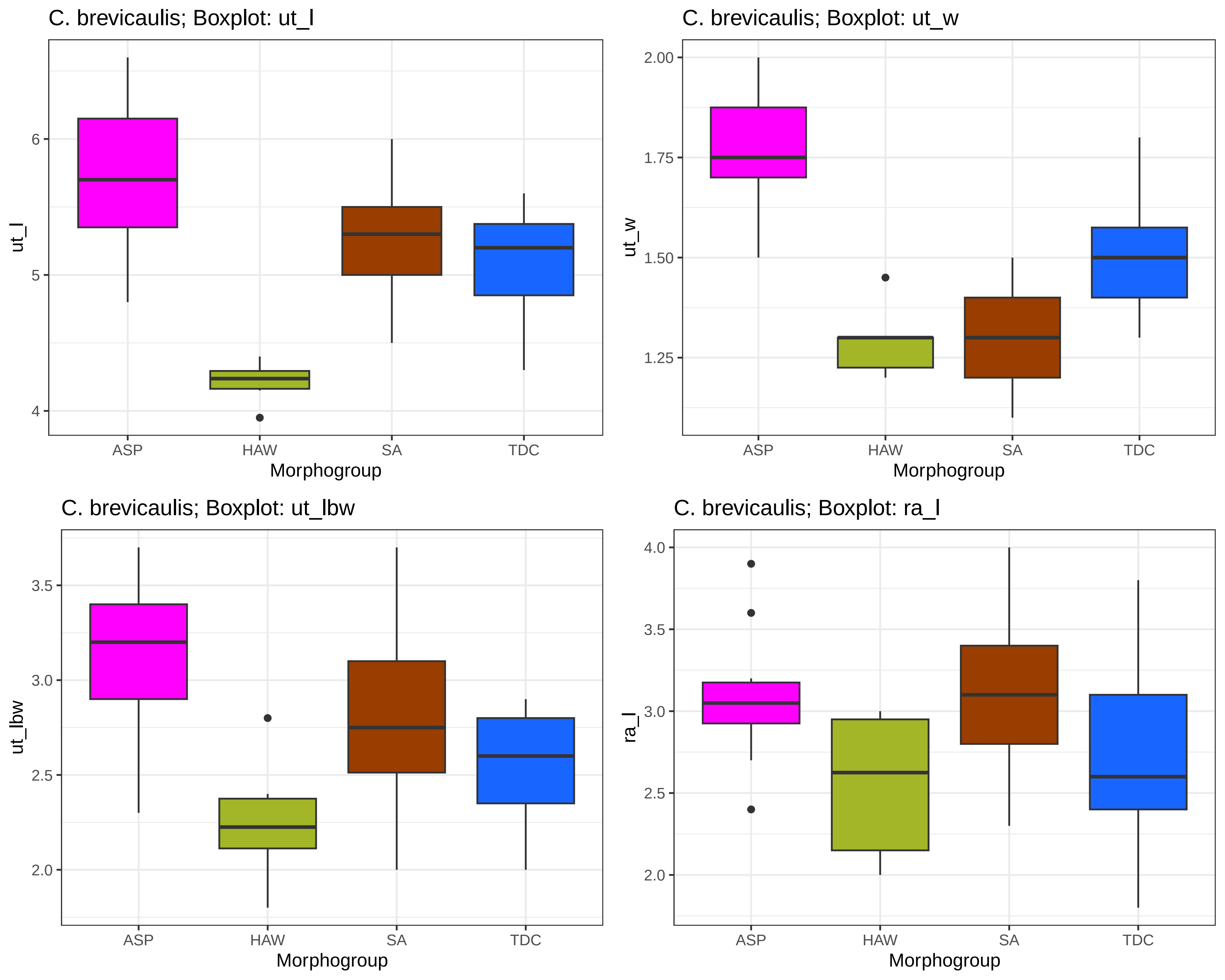
C. brevicaulis; Boxplot: ut_l
ut_l
4
5
6
ASP
HAW
SA
TDC
Morphogroup
C. brevicaulis; Boxplot: ut_w
ut_w
1.25
1.50
1.75
2.00
ASP
HAW
SA
TDC
Morphogroup
C. brevicaulis; Boxplot: ut_lbw
ut_lbw
2.0
2.5
3.0
3.5
ASP
HAW
SA
TDC
Morphogroup
C. brevicaulis; Boxplot: ra_l
ra_l
2.0
2.5
3.0
3.5
4.0
ASP
HAW
SA
TDC
Morphogroup

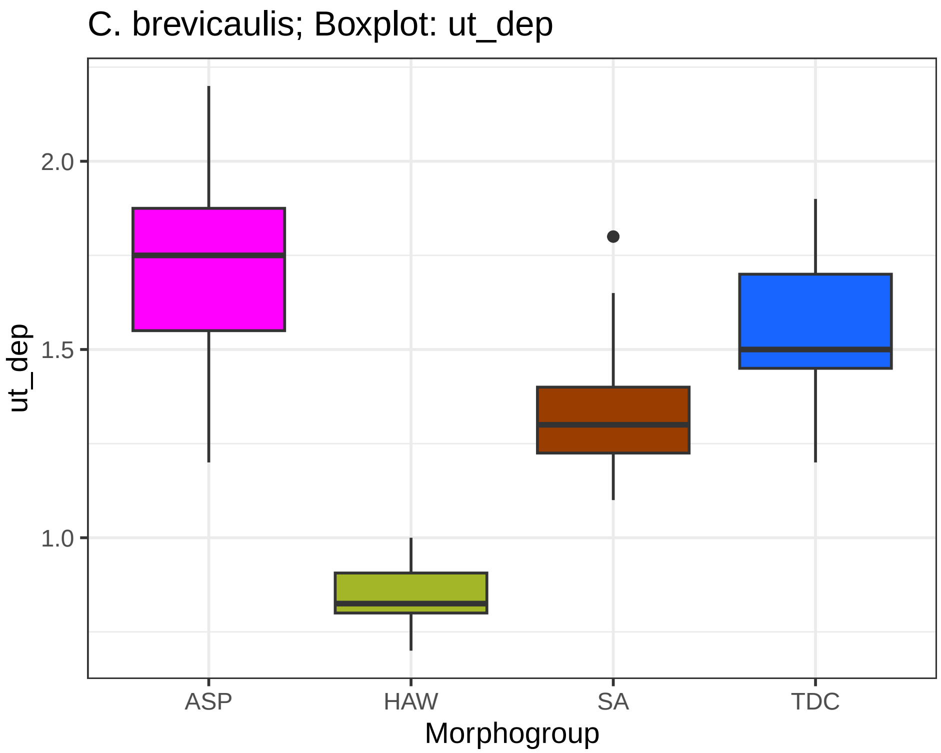
C. brevicaulis; Boxplot: ut_dep
ut_dep
2.0
1.5
1.0
ASP
HAW
SA
TDC
Morphogroup

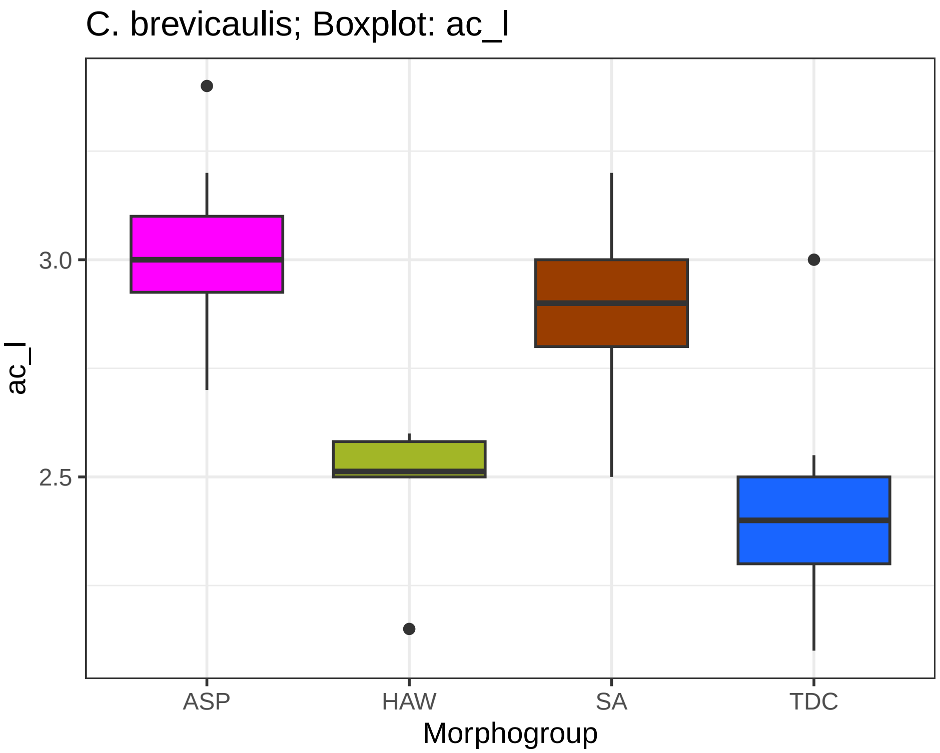
C. brevicaulis; Boxplot: ac_l
ac_l
3.0
2.5
ASP
HAW
SA
TDC
Morphogroup

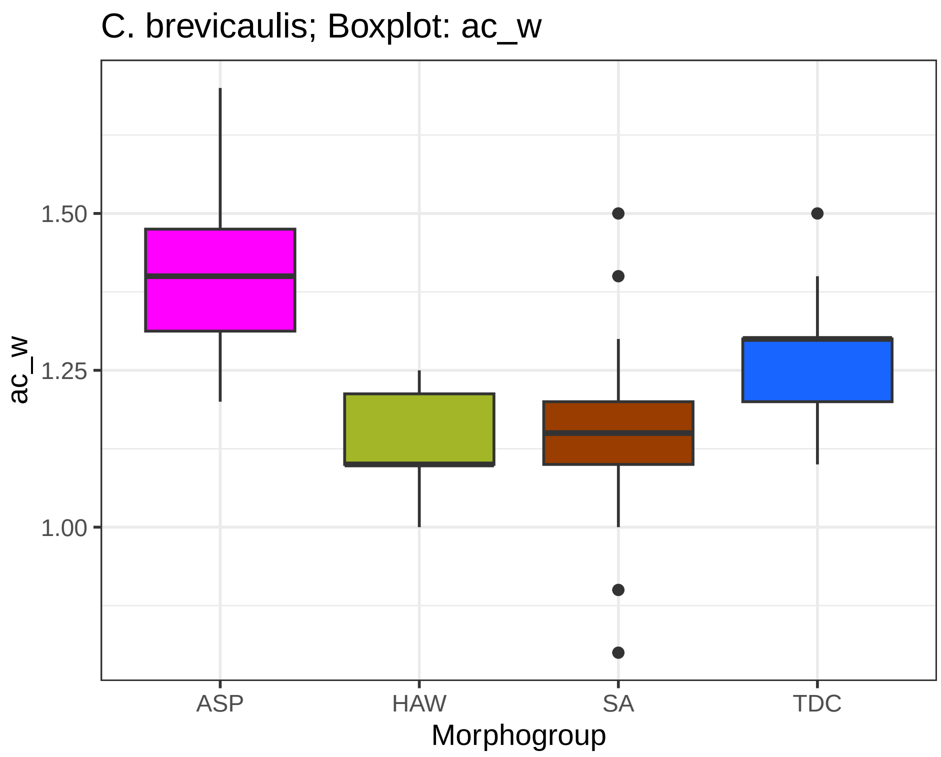
C. brevicaulis; Boxplot: ac_w
ac_w
1.50
1.25
1.00
ASP
HAW
SA
TDC
Morphogroup

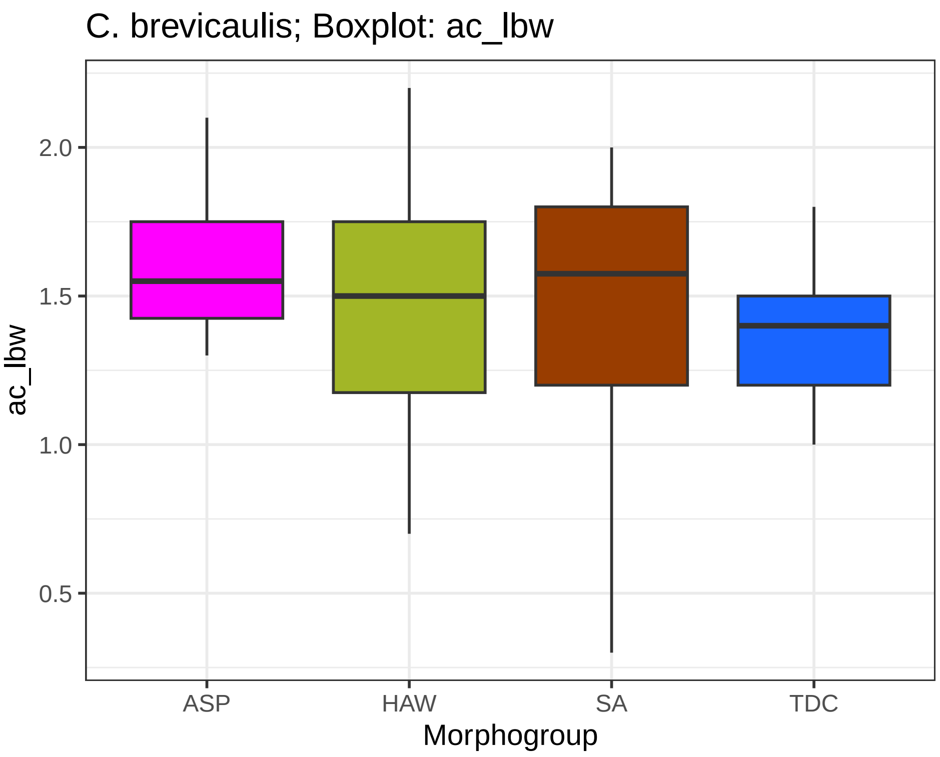
C. brevicaulis; Boxplot: ac_lbw
ac_lbw
2.0
1.5
1.0
0.5
ASP
HAW
SA
TDC
Morphogroup
