# Supplementary material for: Hidden diversity at the edges of maps: morphometrics of Carex sect. Uncinia (Cyperaceae) helps unravel taxonomic diversity in subantarctic and remote archipelagos
Source: PhytoKeys. 2026 Jul 21;277:241–67. doi: 10.3897/phytokeys.277.189029 (PMC13416814; doi:10.3897/phytokeys.277.189029)
Supplement: Supplementary material 2 — Boxplots of all the variables measured in Carex austrocompacta complex [file phytokeys-277-241_article-189029__-s002.pdf]

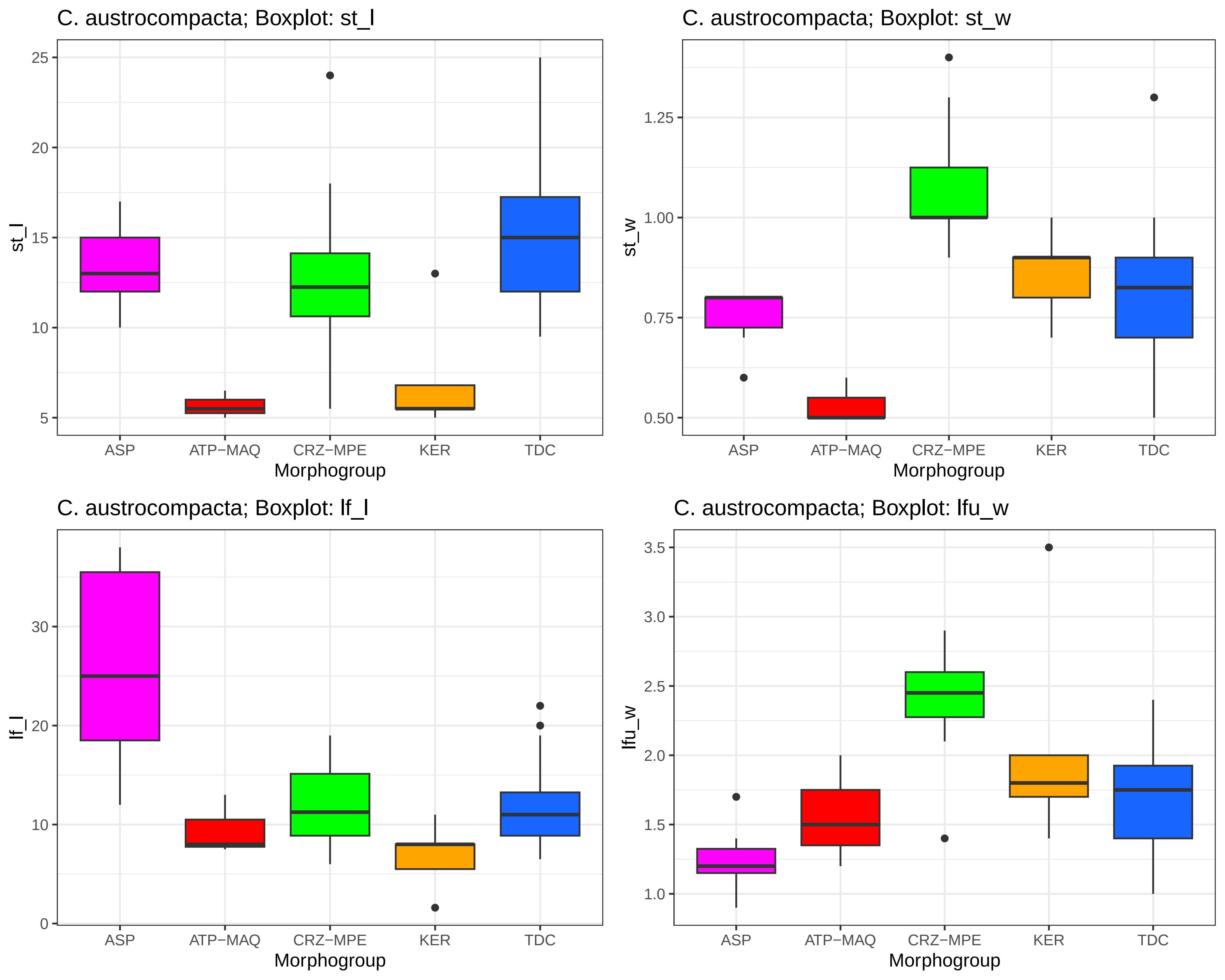
C. austrocompacta; Boxplot: st_l
st_l
25
20
15
10
5
ASP
ATP−MAQ
CRZ−MPE
KER
TDC
Morphogroup
C. austrocompacta; Boxplot: st_w
st_w
1.25
1.00
0.75
0.50
ASP
ATP−MAQ
CRZ−MPE
KER
TDC
Morphogroup
C. austrocompacta; Boxplot: lf_l
lf_l
30
20
10
0
ASP
ATP−MAQ
CRZ−MPE
KER
TDC
Morphogroup
C. austrocompacta; Boxplot: lfu_w
lfu_w
3.5
3.0
2.5
2.0
1.5
1.0
ASP
ATP−MAQ
CRZ−MPE
KER
TDC
Morphogroup

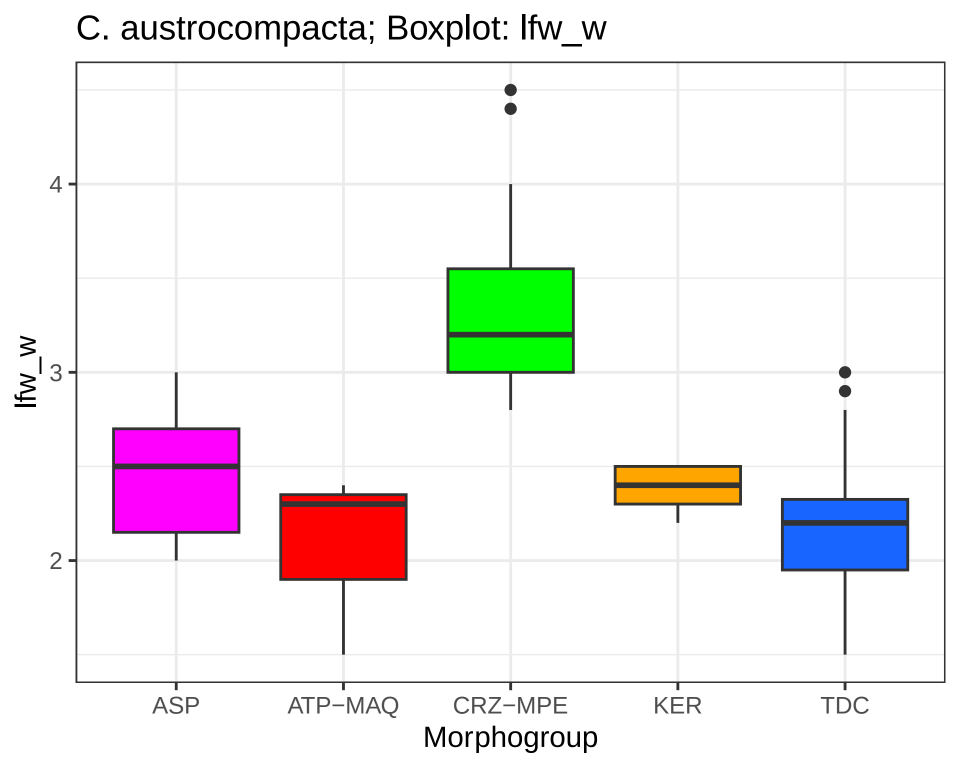
C. austrocompacta; Boxplot: lfw_w
lfw_w
4
3
2
ASP
ATP−MAQ
CRZ−MPE
KER
TDC
Morphogroup

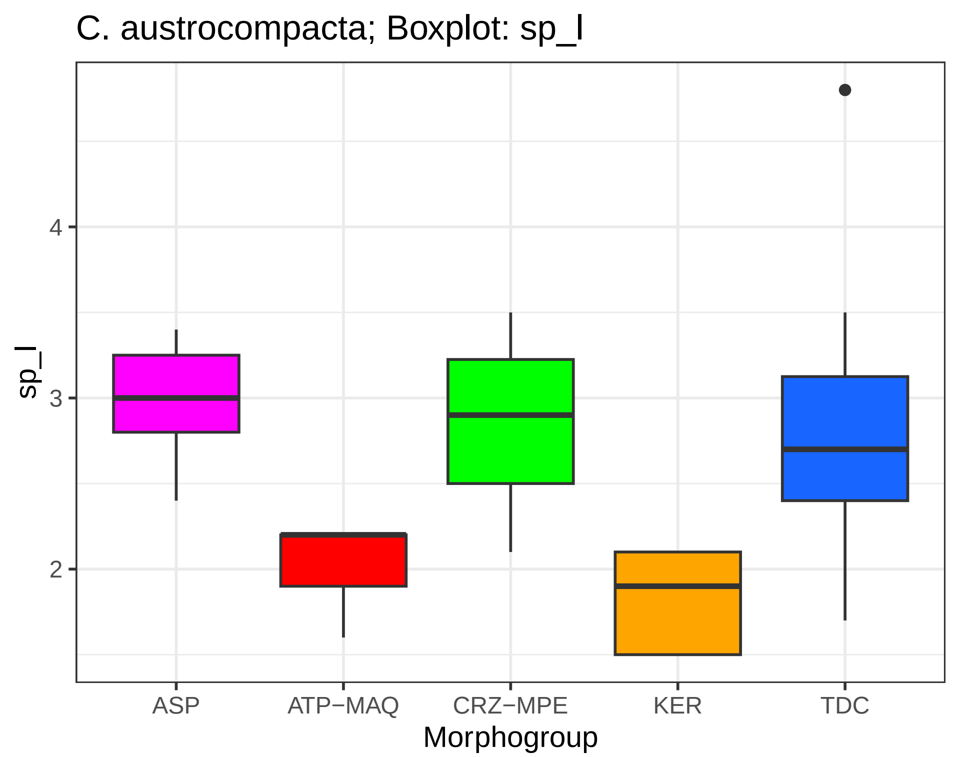
C. austrocompacta; Boxplot: sp_l
sp_l
4
3
2
ASP
ATP−MAQ
CRZ−MPE
KER
TDC
Morphogroup

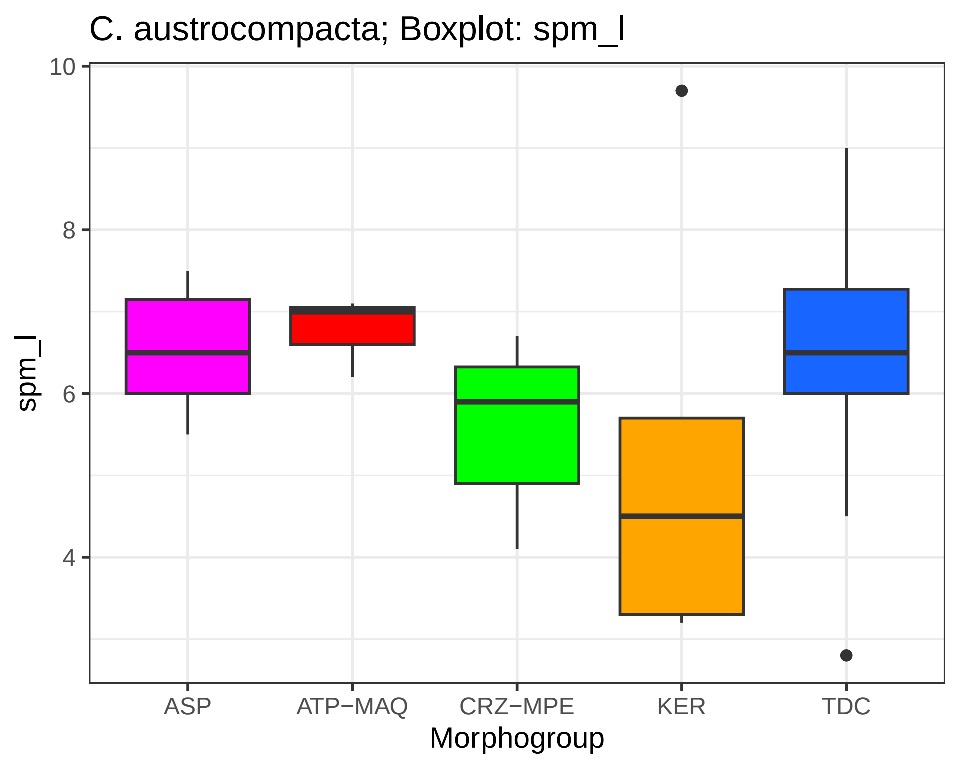
C. austrocompacta; Boxplot: spm_l
spm_l
10
8
6
4
ASP
ATP−MAQ
CRZ−MPE
KER
TDC
Morphogroup

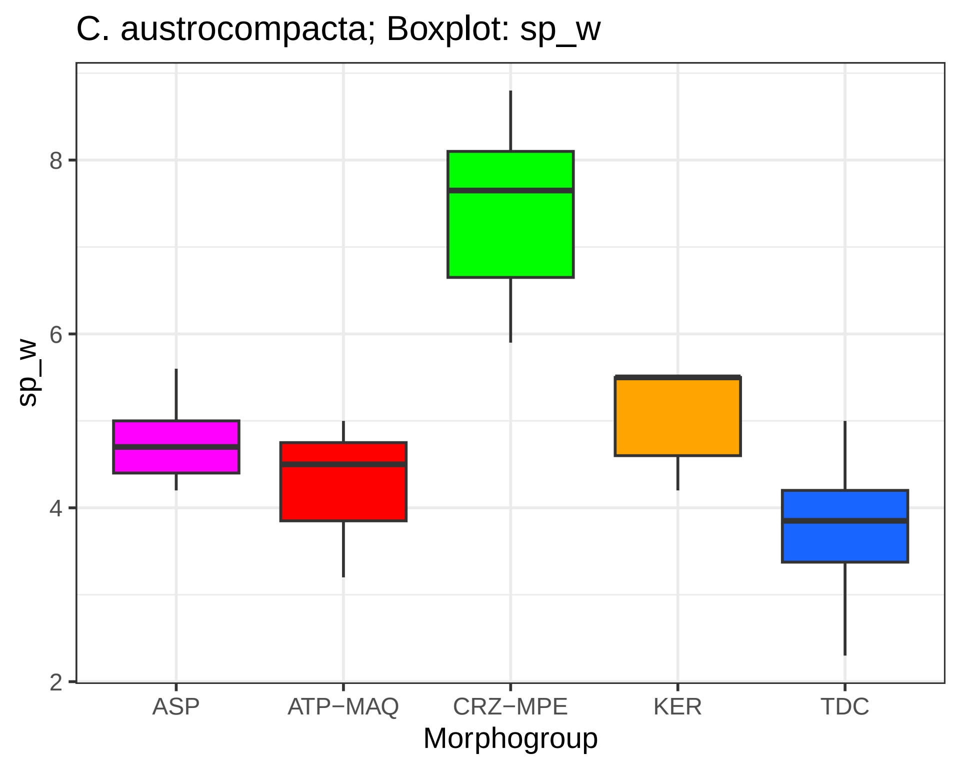
C. austrocompacta; Boxplot: sp_w
sp_w
8
6
4
2
ASP
ATP−MAQ
CRZ−MPE
KER
TDC
Morphogroup

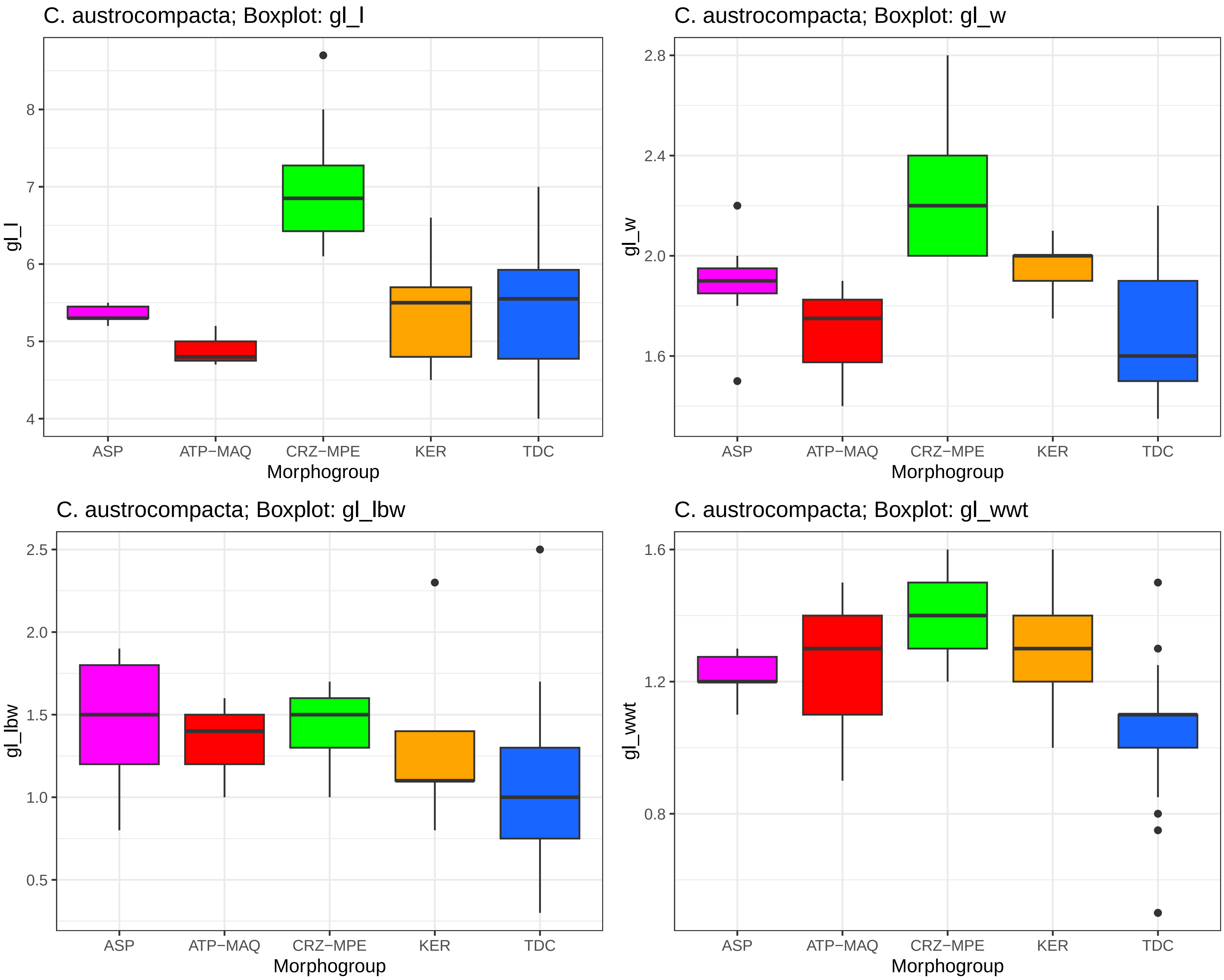
C. austrocompacta; Boxplot: gl_l
gl_l
4
5
6
7
8
ASP
ATP−MAQ
CRZ−MPE
KER
TDC
Morphogroup
C. austrocompacta; Boxplot: gl_w
gl_w
1.6
2.0
2.4
2.8
ASP
ATP−MAQ
CRZ−MPE
KER
TDC
Morphogroup
C. austrocompacta; Boxplot: gl_lbw
gl_lbw
0.5
1.0
1.5
2.0
2.5
ASP
ATP−MAQ
CRZ−MPE
KER
TDC
Morphogroup
C. austrocompacta; Boxplot: gl_wwt
gl_wwt
0.8
1.2
1.6
ASP
ATP−MAQ
CRZ−MPE
KER
TDC
Morphogroup

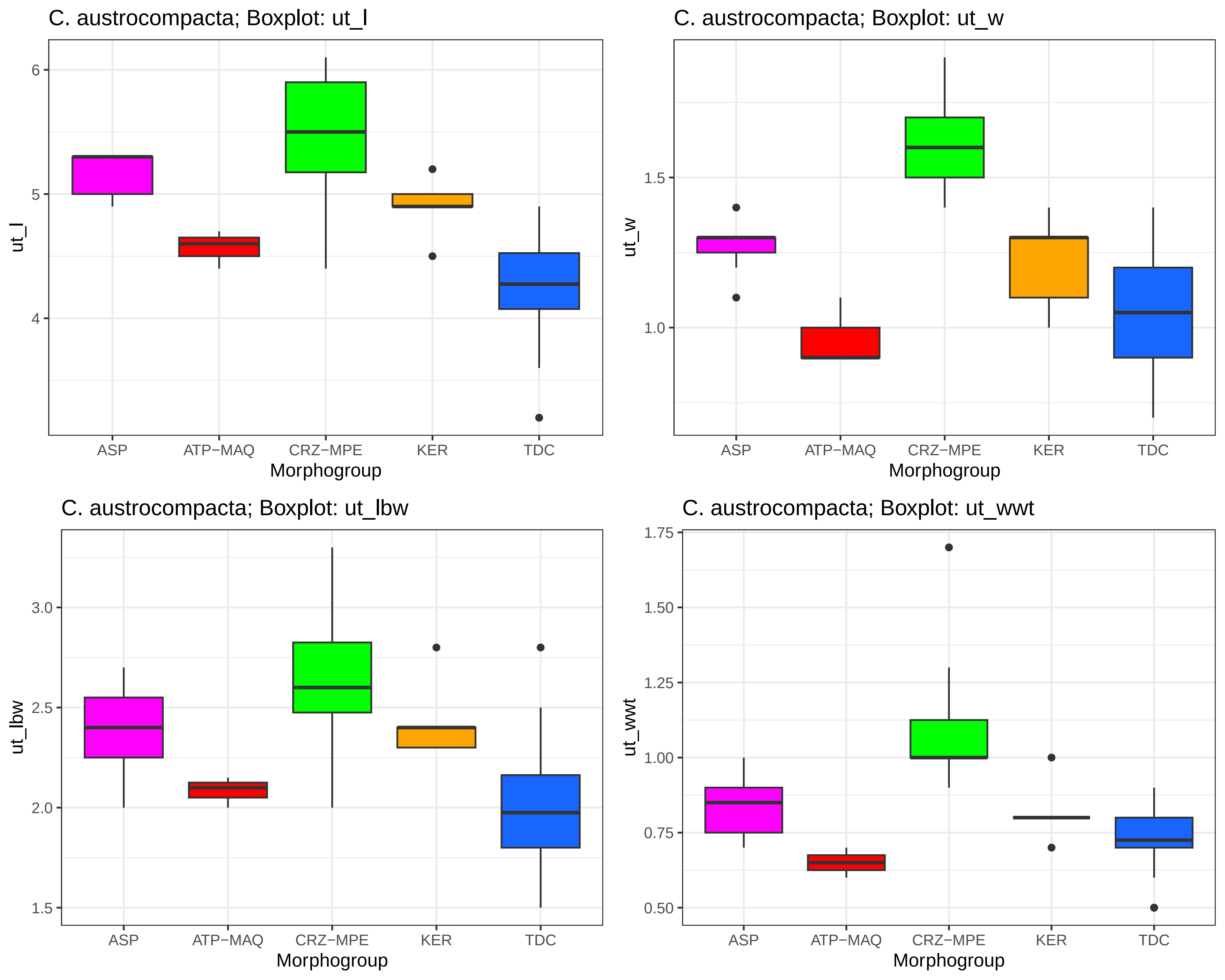
C. austrocompacta; Boxplot: ut_l
ut_l
6
5
4
ASP
ATP−MAQ
CRZ−MPE
KER
TDC
Morphogroup
C. austrocompacta; Boxplot: ut_w
ut_w
1.5
1.0
ASP
ATP−MAQ
CRZ−MPE
KER
TDC
Morphogroup
C. austrocompacta; Boxplot: ut_lbw
ut_lbw
3.0
2.5
2.0
1.5
ASP
ATP−MAQ
CRZ−MPE
KER
TDC
Morphogroup
C. austrocompacta; Boxplot: ut_wwt
ut_wwt
1.75
1.50
1.25
1.00
0.75
0.50
ASP
ATP−MAQ
CRZ−MPE
KER
TDC
Morphogroup

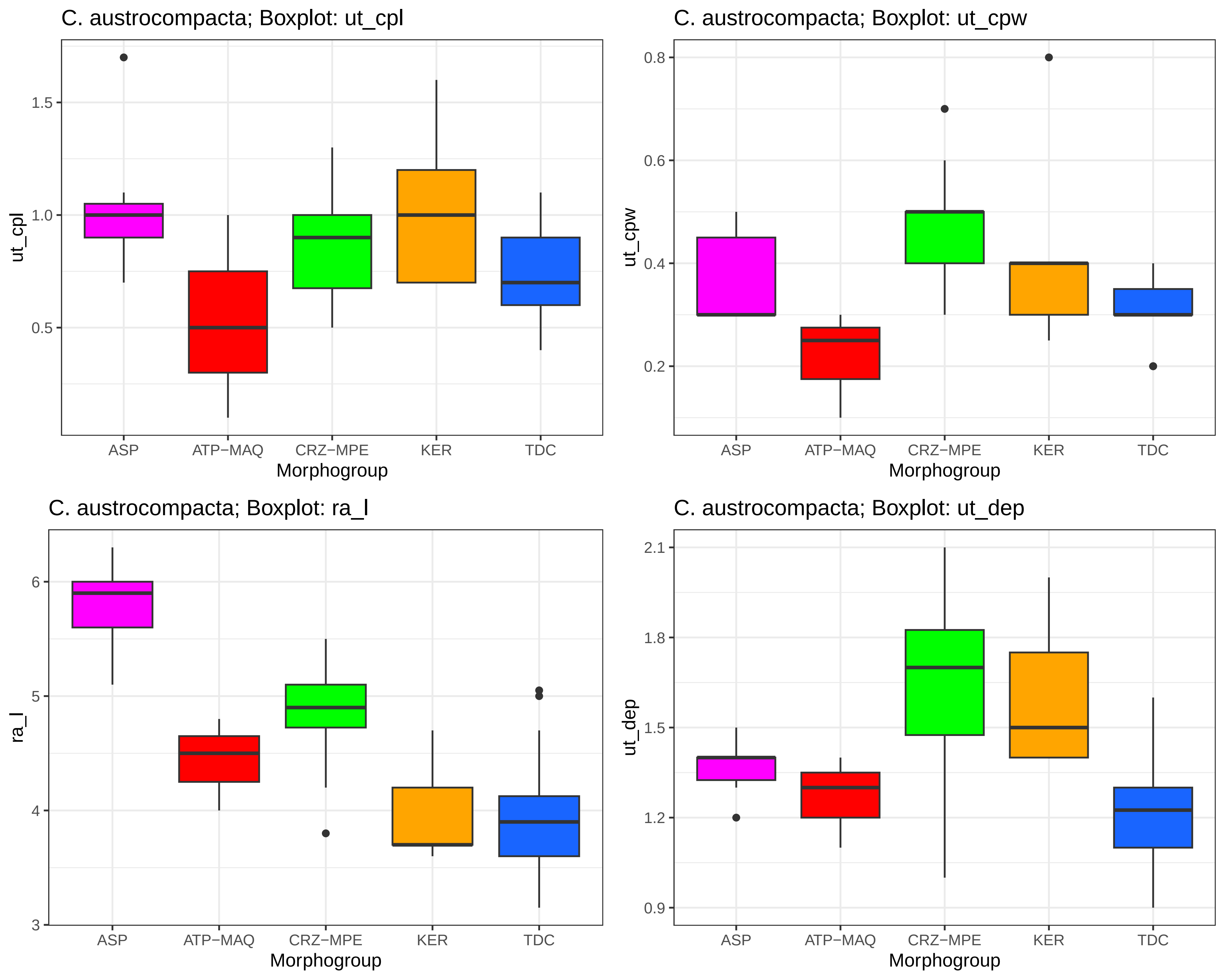
C. austrocompacta; Boxplot: ut_cpl
ut_cpl
1.5
1.0
0.5
ASP
ATP−MAQ
CRZ−MPE
KER
TDC
Morphogroup
C. austrocompacta; Boxplot: ut_cpw
ut_cpw
0.8
0.6
0.4
0.2
ASP
ATP−MAQ
CRZ−MPE
KER
TDC
Morphogroup
C. austrocompacta; Boxplot: ra_l
ra_l
6
5
4
3
ASP
ATP−MAQ
CRZ−MPE
KER
TDC
Morphogroup
C. austrocompacta; Boxplot: ut_dep
ut_dep
2.1
1.8
1.5
1.2
0.9
ASP
ATP−MAQ
CRZ−MPE
KER
TDC
Morphogroup

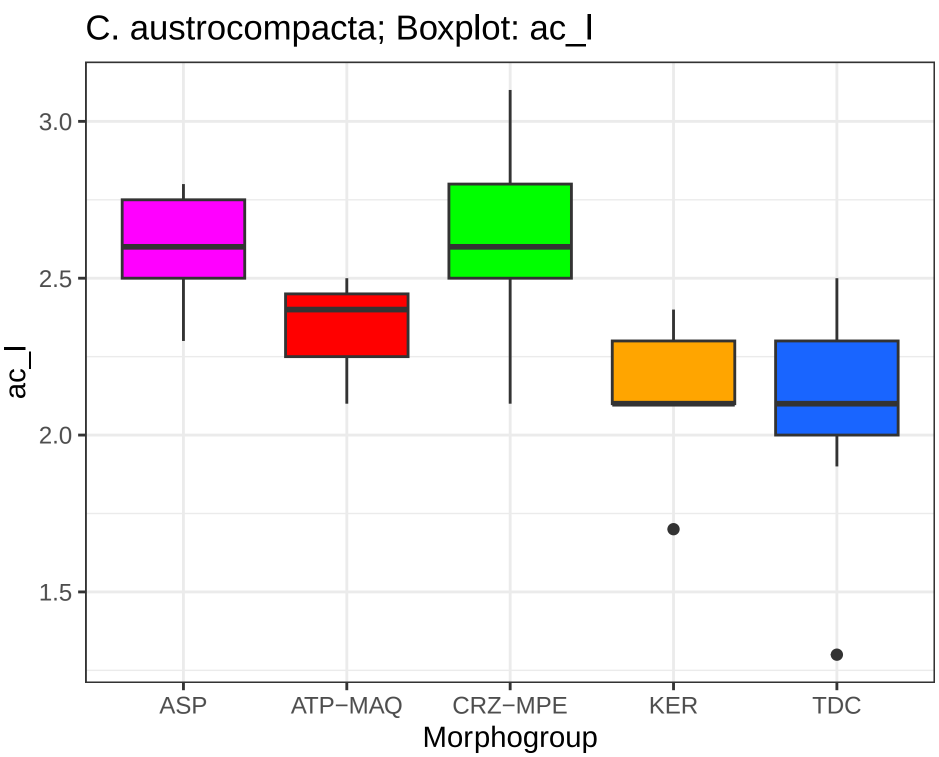
C. austrocompacta; Boxplot: ac_l
ac_l
3.0
2.5
2.0
1.5
ASP
ATP−MAQ
CRZ−MPE
KER
TDC
Morphogroup
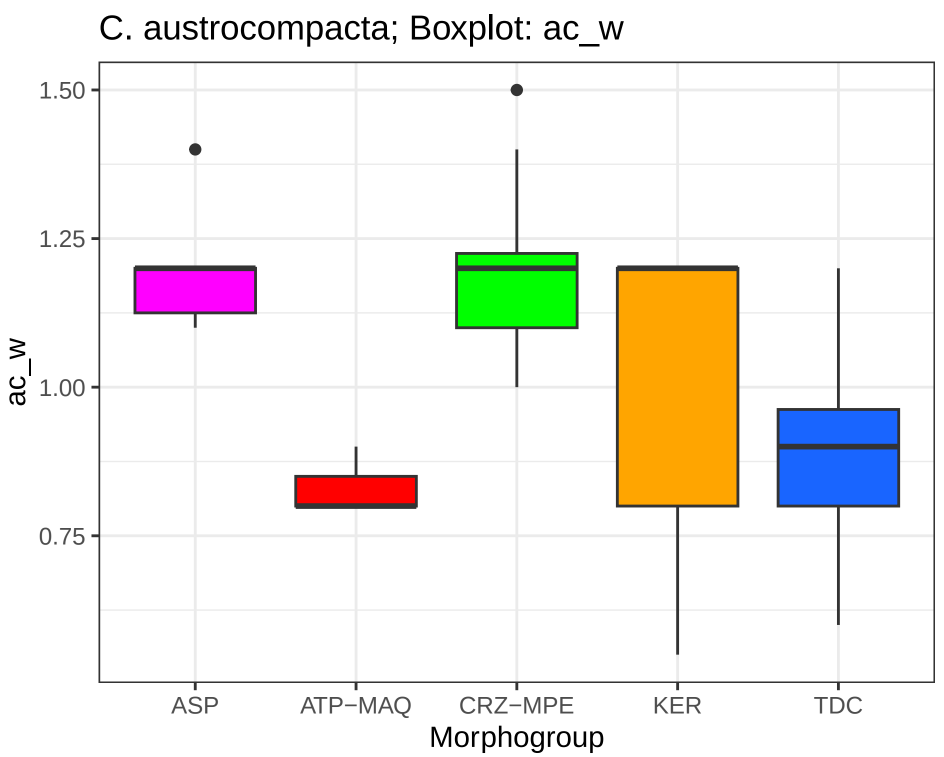
C. austrocompacta; Boxplot: ac_w
ac_w
1.50
1.25
1.00
0.75
ASP
ATP−MAQ
CRZ−MPE
KER
TDC
Morphogroup
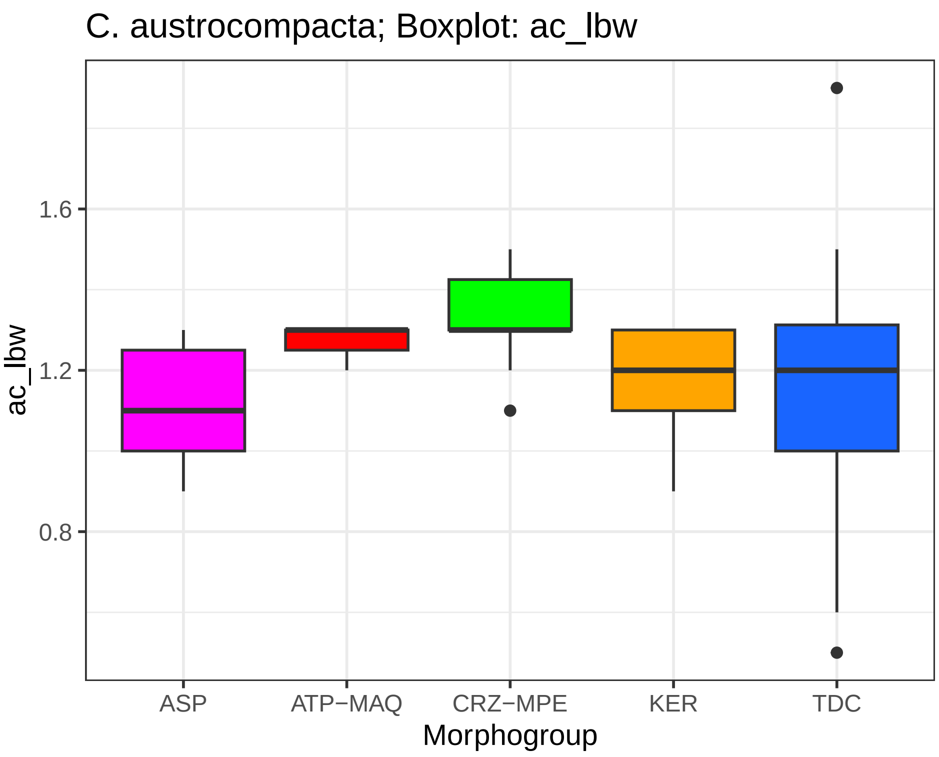
C. austrocompacta; Boxplot: ac_lbw
ac_lbw
1.6
1.2
0.8
ASP
ATP−MAQ
CRZ−MPE
KER
TDC
Morphogroup
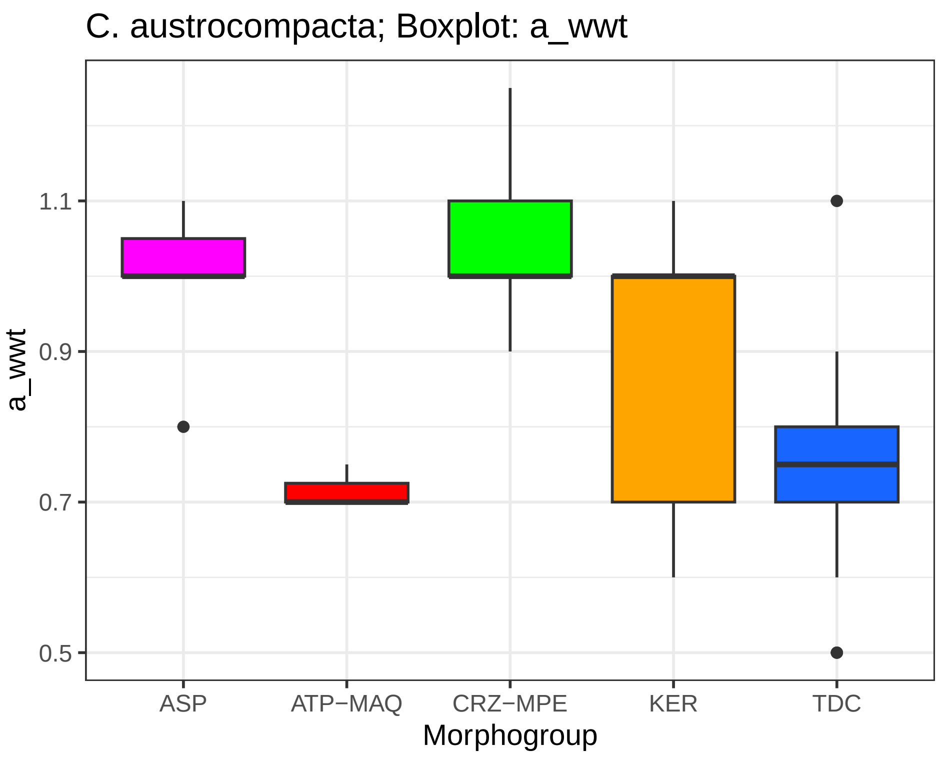
C. austrocompacta; Boxplot: a_wwt
a_wwt
1.1
0.9
0.7
0.5
ASP
ATP−MAQ
CRZ−MPE
KER
TDC
Morphogroup

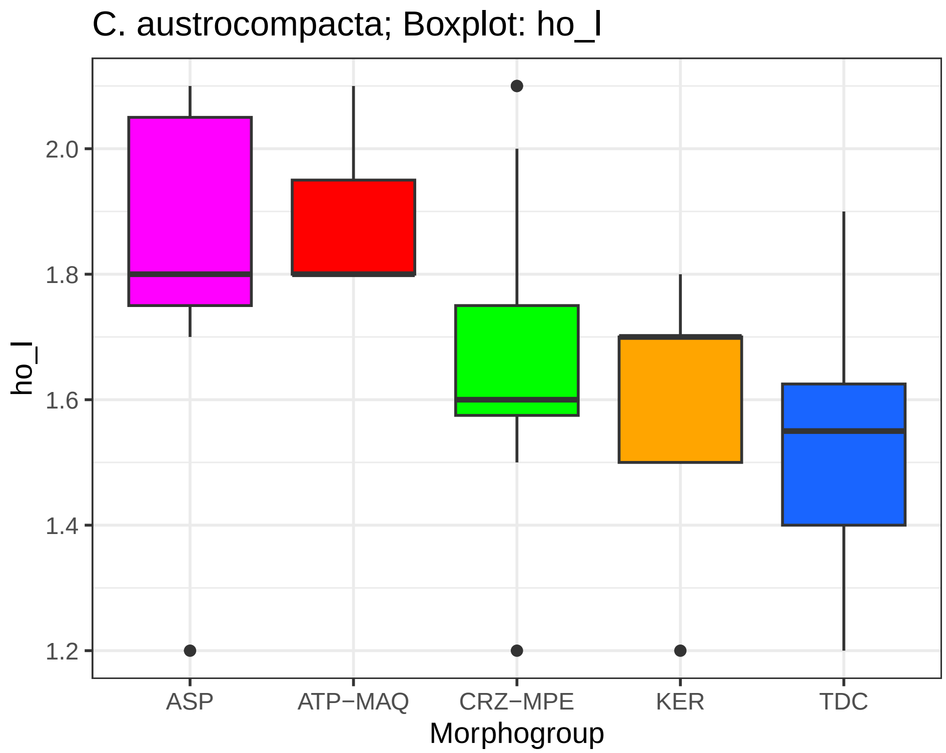

C. austrocompacta; Boxplot: ho_l
ho_l
2.0
1.8
1.6
1.4
1.2
ASP
ATP−MAQ
CRZ−MPE
KER
TDC
Morphogroup
